# Seasonal hydrochemical characteristics of spring water in Southern Poland: integrating geochemical modeling, health risk analysis and mitigation strategies

**DOI:** 10.1038/s41598-025-10322-5

**Published:** 2025-07-15

**Authors:** Mohamed Hamdy Eid, Omar Saeed, Péter Szűcs, Marek Ruman, Dominika Dąbrowska, Vahid Nourani

**Affiliations:** 1 Faculty of Earth Science, Institute of Environmental Management, University of Miskolc, Miskolc-Egyetemváros, 3515 Hungary; 2 Geology Department, Faculty of Science, Beni-Suef University, Beni-Suef, 65211 Egypt; 3 Doctoral School of Environmental Science, Hungarian University of Agriculture and Life Sciences (MATE), Páter Károly u. 1, Gödöllő, 2100 Hungary; 4 Faculty of Natural Sciences, University of Silesia, Będzińska 60, 41-200 Sosnowiec, Poland; 5 Faculty of Civil Engineering, Center of Excellence in Hydroinformatics, University of Tabriz, Tabriz, 166616471 Iran; 6 Faculty of Civil and Environmental Engineering, Near East University, Via Mersin 10, Istanbul, Turkey

**Keywords:** Seasonal water chemistry, Hydrogeochemical modeling, Canadian water quality index, Health risk, Corrosion potential, Monte Carlo simulation, Environmental sciences, Risk factors

## Abstract

This study investigates seasonal hydrochemical characteristics of spring water at six sites in southern Poland (Leśniów, Zygmunt, Halszka, Dobro Woda, Święto Woda, and Zimny Sztok) using integrated geochemical modeling, health risk assessment, and water quality indexing. Seasonal sampling revealed distinct temporal patterns in major ion concentrations. Calcium and magnesium concentrations were significantly higher during winter (Ca^2+^: 90–96 mg/L; Mg^2+^: 5.1–7.2 mg/L) compared to summer (Ca^2+^: 19–45 mg/L; Mg^2+^: 3–5 mg/L), attributed to reduced biological uptake and enhanced carbonate dissolution at lower temperatures. Conversely, sodium (2.5–11 mg/L) and chloride (13–28 mg/L) concentrations peaked during summer due to evaporative concentration and anthropogenic influences. Heavy metals (Fe, Mn, Hg) showed maximum concentrations in summer. Hydrogeochemical analysis identified two water types: Ca–Mg–HCO_2_ and Ca–Mg–Cl/SO_4_. PHREEQC modeling revealed undersaturation in calcite (−4.27 to 0.1), dolomite (−9.08 to −1.25), and gypsum (−2.6 to −1.78). Canadian Water Quality Index (CWQI) values (84.57–96.52) classified all samples as “Good,” while Heavy Metal Pollution Index (HPI) values (20.27–120.10) and Metal Index (MI) values (0.58–2.35) indicated highest contamination at Zimny Sztok and Leśniów. Ecological Risk Index (ERI) values (0.18–4.17) suggested low ecological risk. Health risk assessment demonstrated children face 1.5−2× higher non-carcinogenic risks than adults, with maximum hazard index (HI) of 3.37 at Leśniów, primarily from mercury exposure with hazard quotient (HQ) = 3.01. Corrosion indices indicated predominantly corrosive conditions with minimal scaling potential.

**Supplementary Information:**

The online version contains supplementary material available at 10.1038/s41598-025-10322-5.

## Introduction

Water security and quality represent fundamental global challenges of the 21st century, with far-reaching implications for human health, environmental sustainability, and socioeconomic development^1–3^. Access to good quality water is essential for maintaining public health and ecosystem integrity, yet this vital resource faces unprecedented threats worldwide^4–7^. The escalating pressures of population growth, industrial expansion, and climate change have intensified water quality degradation across diverse geographical contexts, from densely populated urban centers to remote rural landscapes^8–11^. This global water crisis manifests through multiple contaminant pathways, including heavy metals, microplastics, agricultural chemicals, and organic pollutants that migrate through soil matrices into groundwater systems^7,12–14^.

Within this global context, groundwater resources merit special attention as they constitute approximately 30% of the world’s freshwater supply and serve as the primary drinking water source for nearly half the global population^15^. A spring is a natural source of water that is formed when a flowing or moving mass of groundwater at or below the local groundwater table crosses the side of a valley or the bottom^16^. Nowadays, although spring waters have lost their importance in the context of the basic water supply of the population, they are a valuable element of the landscape, a link between the surface and groundwater systems and as an additional source of water supply associated with health properties^8,17,18^. The protection of spring water resources is extremely important because the contamination of spring water can also affect the contamination of groundwater. Faults, fractures or water-rock contact are the ways in which springs come to the surface^19^. The presence of springs is influenced by, among others, lithological formation, precipitation level and rock characteristics and the main factors determining physicochemical properties are land use, geology and climate^1,6,8,20^. One example of backup sources of drinking water is karst systems^21^. It should be noted that they provide about 9% of the world’s drinking water. Furthermore, karst aquifer systems, are great locations to track changes in pollutant concentrations and the rate of migration. Spring waters contain minerals from the surrounding rocks, but they are also vulnerable to chemical changes brought on by atmospheric precipitation. This is because the hydrology and ecology of springs are closely linked to changes in the interactions of surface and groundwater^22–24^. Many small springs are located within valuable natural areas or urban recreational areas. The water quality in such springs, however, remains largely unknown due to the fact that they are not included in national monitoring networks. The transition from global water quality concerns to regional challenges requires consideration of specific geographical contexts^25^. In Poland, particularly its southern regions, water resource management faces unique challenges stemming from the intersection of historical industrial activities, geological complexity, and evolving regulatory frameworks.

Understanding the geochemical behavior of water requires consideration of key factors including recharge dynamics, aquifer properties, water-rock interaction duration, and specific geochemical processes such as mineral dissolution, precipitation, and ion exchange^1,26–30^. Recent advances in hydrogeochemical assessment have employed integrated analytical approaches combining traditional techniques with advanced statistical methods and geospatial analysis. Multivariate statistical techniques including correlation analysis, principal component analysis (PCA), and hierarchical cluster analysis (HCA) have emerged as powerful tools for interpreting complex water quality datasets and identifying underlying factors controlling hydrochemical variations^31,32^. These approaches, when combined with graphical methods such as Piper diagrams, Gibbs plots, and Chadha diagrams, provide comprehensive frameworks for characterizing water types, identifying geochemical processes, and distinguishing natural versus anthropogenic influences on water chemistry^28,30,33–35^. Additionally, geochemical modeling provides a powerful way to simulate and predict chemical reactions within aquifer systems, such as the dissolution or formation of minerals, ion exchange, and the adsorption of substances by clay minerals^28,30,36,37^.

The complexity of water quality assessment involving numerous parameters, multiple sampling locations, and temporal variations has driven the development of integrative indices that distill multidimensional data into more accessible metrics^25,32^. The Water Quality Index (WQI) approach, which has gained widespread acceptance since its introduction by Horton^38^provides a standardized method for evaluating overall water quality status through a single numerical value. Recent refinements to WQI methodologies have enhanced their applicability across diverse environmental contexts, with the Canadian Water Quality Index (CWQI) emerging as a particularly robust framework due to its ability to incorporate multiple parameters and account for both the frequency and magnitude of guideline exceedances^14,39^. Contemporary applications of WQI in coal mining regions have demonstrated its utility for identifying spatial patterns in water quality and prioritizing areas for remediation efforts^40^.

Heavy metal contamination represents a significant concern in many groundwater systems due to the potential health risks associated with chronic exposure. Recent health risk assessment frameworks have evolved beyond traditional deterministic approaches to incorporate probabilistic methods that better account for uncertainty and variability in exposure parameters^4–6,41^. Monte Carlo simulations and sensitivity analyses using Sobol indicators have emerged as valuable tools for quantifying uncertainty in risk estimates and identifying the most influential parameters driving health risks^10,18,42,43^. These advanced approaches provide more nuanced understanding of exposure pathways and potential health impacts, particularly for vulnerable populations such as children who often face disproportionate risks due to physiological and behavioral factors^44^.

One of the key challenges in water management is understanding and mitigating the corrosive potential of water, which can lead to significant damage to pipelines, storage systems, and other infrastructure. Corrosion is influenced by a complex interplay of chemical parameters, including calcium carbonate saturation, chloride-sulfate ratios, and buffering capacity, among others. This study evaluates the corrosivity and scaling potential of water samples using well-established indices such as the Langelier Saturation Index (LSI), Ryznar Stability Index (RSI), Aggressive Index (AI), Puckorius Scaling Index (PSI), Revelle Index (RI), Chloride-Sulfate Mass Ratio (CSMR), and Larson-Skold Index (LS). These indices provide insights into the chemical stability of water, its tendency to corrode or form scales, and its susceptibility to salinization and pH changes^45–47^.

Despite significant advances in water quality assessment methodologies, important research gaps remain, particularly regarding the integration of multiple analytical approaches to develop comprehensive understanding of water quality dynamics in complex environmental settings. While numerous studies have examined individual aspects of water quality in southern Poland, few have implemented integrated approaches combining geochemical modeling, health risk assessment, and water quality indices specifically for spring water systems in this region. This knowledge gap is particularly significant given the continued use of springs as supplementary water sources and their potential as early warning indicators for broader groundwater quality trends.

This study addresses these research gaps through a comprehensive assessment of spring water quality at six locations in southern Poland, employing an integrated methodological framework not previously applied in this study area. The novelty of this research lies in its multifaceted approach that combines: (i) geochemical modeling to characterize water-rock interactions and mineral saturation states; (ii) application of diverse graphical methods and ionic ratios to differentiate natural versus anthropogenic influences; (iii) implementation of advanced probabilistic methods for environmental, ecological, and age-specific health risk assessment; (iv) application of the Canadian Water Quality Index to synthesize multiple water quality parameters; and (v) evaluation of corrosion potential using multiple complementary indices. This integrated approach provides a more holistic understanding of spring water quality dynamics than previous single-method studies, offering valuable insights for water resource management and public health protection in the region. The findings have significant implications for decision-makers and water management planning, as they represent the first comprehensive investigation of these specific challenges in southern Poland using this integrated methodological framework.

## Materials and methods

### Study area description

The study was conducted for six springs located in the southern part of Poland. Three of them are located within the Kraków-Częstochowa Upland, and the remaining three within the Silesian Upland.

**Fig. 1:**
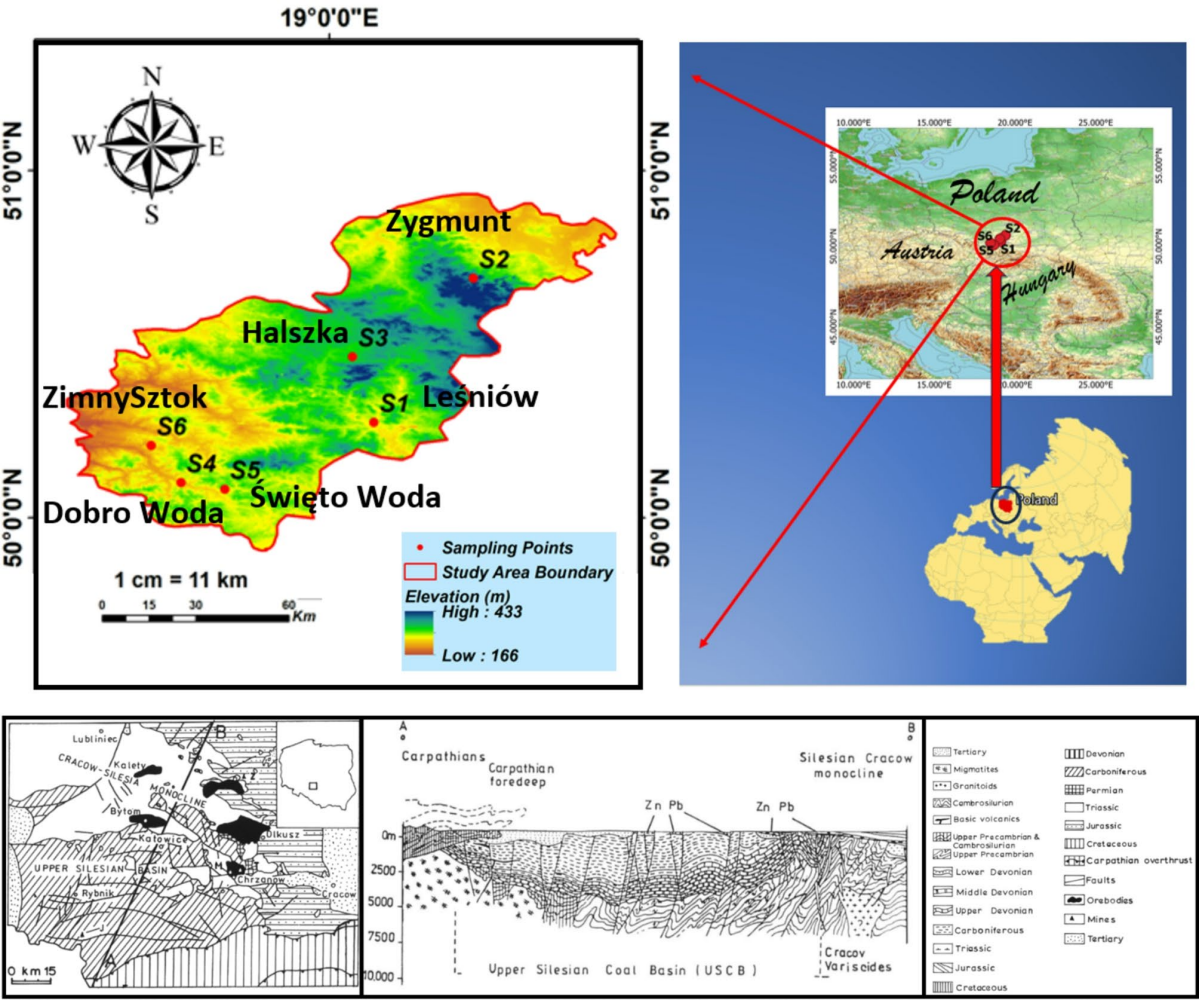
Sampling sites, geological map and cross section of the study area in southern Poland^54^.

The first spring is located in Leśniów (Fig. 1). It is found within the Żarki commune (southern Poland) at a height of 345 m above sea level. In terms of physical and topographical regionalization, according to Dąbrowska^48^, it is found within the Kraków-Częstochowa Upland macroregion and the Częstochowa Upland mesoregion. The spring is encased with taps installed at the outlet for water collection. Due to the fact that the spring is found within the range of the sanctuary, travelers and sightseers collect the water. The water streams out through a stone trough.

The second spring is the biggest outflow in the system of the Zygmunt Spring, which is found within the Janów commune with an altitude of 296 m above sea level. The spring is found within the Jurassic level and forms the headwaters of the Wiercica catchment. It is a perennial spring. Water from the spring makes the Zygmunt Stream, which is 500 m long. The most important source of water supply for the residents and tourists regularly collect water from the spring in Złoty Potok is taken all the time by adjacent inhabitants and sightseers. The near vicinity of the street (approximately 150 m) makes the location accessible.

The next studied source is the Halszka spring located in Sokolniki, Niegowa commune. In terms of physiogeography^17,48^ the commune is located within the Częstochowa Upland mesoregions. The spring is located at an altitude of 262 m above sea level, i.e. in the lowest part of the commune in the Halszka river valley.

The second group consists of springs located in recreational areas - i.e. the Dobro Woda spring, Święto Woda and Zimny Sztok. The first two are located in the so-called Palowice Lakeland in the Silesian Upland macroregion and the Katowice Upland mesoregion. This anthropogenic lakeland consists of 10 breeding ponds and three post-mining reservoirs with a total area of 57 ha^49^. This area was heavily industrialized in the past.

The Zimny Sztok spring is located within the macroregion of the Silesian Upland and the mesoregion of the Rybnik Plateau at an altitude of 224.6 m above sea level.

The research area is situated within the warm and transitional temperate climate zone. The average annual air temperature fluctuates between 7 and 8 degrees Celsius, with an annual amplitude ranging from 21 to 23 degrees Celsius. The mean yearly precipitation varies between 700 and 750 mm, contingent upon the topographical features and land surface characteristics. The highest amount of monthly precipitation is recorded in July and August, while the lowest occurs in January and February. The maximum relative air humidity value is attained during the winter season, reaching up to 85%, while the minimum, approximately 70%, is observed in the summer months^17,49–51^.

### Geology and Hydrogeology of the area

The study area encompasses two primary geological units. The Leśniów springs (Zygmunt and Halszka) are located within the Silesian-Cracow monocline, characterized by Paleozoic folded formations (Cambrian to Carboniferous) overlain by Mesozoic epicontinental deposits, including Triassic sandstones, gypsum-bearing mudstones, and carbonates, as well as Jurassic limestone layers exceeding 500 m in thickness. Quaternary deposits consist of fluvial-glacial sands and gravels. In contrast, the Dobro Woda, Święto Woda, and Zimny Sztok springs lie within the Upper Silesian Block, featuring Precambrian crystalline basement rocks (mica schists and paragneisses) overlain by Cambrian siltstones and diabases, Devonian carbonates, and Carboniferous clastic and coal-bearing sequences. These are capped by Miocene Carpathian Foredeep deposits (clays, sands, and gypsum/anhydrite) and Quaternary glacial sediments. The aquifer systems vary by location: karst springs are fed by three aquifers (Quaternary sands/gravels, Tertiary sands/cobbles, and Jurassic limestone), while other springs rely on two aquifers (Quaternary fluvio-glacial deposits and Neogene clays) (Fig. 1)^49,52,53^.

### Sampling and analysis

A total of 23 water samples were collected from six spring locations in southern Poland during four seasonal sampling campaigns (November, February, May, and August, 2024) to assess temporal and spatial variations in water quality. The sampling sites included three springs located within the Kraków-Częstochowa Upland (Leśniów, Zygmunt, and Halszka) and three springs within the Silesian Upland (Dobro Woda, Święto Woda, and Zimny Sztok). All sampling was conducted following standardized protocols to ensure data reliability and comparability across seasons.

Field measurements were performed using calibrated Elmetron instruments that were verified immediately before each sampling event. Temperature, electrolytic conductivity (EC), pH, and dissolved oxygen were measured in-situ using a CC-401 conductivity meter (range: 0-1000 mS/cm, accuracy: ± 0.01 µS/cm), a CP-411 pH meter (range: 0–14, accuracy: ± 0.01 pH units), and a CO-401 oxygen meter (range: 0–20 mg/L, accuracy: ± 0.01 mg/L), respectively. Spring discharge was also measured at each location.

Water samples for laboratory analysis were collected in pre-cleaned polyethylene bottles provided by the GBA Polska accredited laboratory (accreditation number: AB 1095). For each sampling point, duplicate samples were collected to allow for quality control and potential reanalysis. Samples for metal analysis were preserved with ultra-pure nitric acid (pH < 2) and stored at 4 °C during transport to the laboratory. All samples were processed within 24 h of collection to minimize potential changes in water chemistry.

Chemical analyses were performed using an Agilent 7900 ICP-MS (Agilent Technologies, Santa Clara, CA, USA) for metal determination and a Metrohm 883 Basic IC plus ion chromatograph (Metrohm AG, Herisau, Switzerland) for anion analysis. The ICP-MS system was equipped with an octopole reaction system (ORS) with helium collision mode for interference removal, a high matrix introduction (HMI) system, a concentric nebulizer, and a quartz torch with 2.5 mm internal diameter. The ion chromatograph was fitted with a Metrosep A Supp 5 column (250 × 4.0 mm) for anion separation. These instruments were selected for their high sensitivity and ability to detect trace concentrations of analytes in environmental water samples.

A comprehensive suite of 26 physicochemical parameters was analyzed, including major ions (Ca^2+^, Mg^2+^, Na^+^, K^+^, HCO_2_^−^, Cl^−^, NO_2_^−^, SO_4_^2−^), trace metals (Fe, Al, Mn, Ni, Cu, Sr, Cd, Cr, Pb, Hg, Zn), and general parameters (pH, EC, acidity, alkalinity). Method detection limits (MDLs) were determined according to EPA Method 200.8 by analyzing seven replicates of a low-level standard and multiplying the standard deviation by 3.14 (99% confidence level). The MDLs for major ions were: Ca^2+^ (0.005 mg/L), Mg^2+^ (0.003 mg/L), Na^+^ (0.5 mg/L), K^+^ (0.5 mg/L), HCO_2_^−^ (5.0 mg/L), Cl^−^ (2.0 mg/L), NO_2_^−^ (0.4 mg/L), and SO_4_^2−^ (5.0 mg/L). For trace metals, the MDLs were: Fe (0.002 mg/L), Al (0.005 mg/L), Mn (0.0005 mg/L), Ni (0.002 mg/L), Cu (0.002 mg/L), Sr (0.001 mg/L), Cd (0.0001 mg/L), Cr (0.0005 mg/L), Pb (0.001 mg/L), Hg (0.00005 mg/L), and Zn (0.002 mg/L). These detection limits were sufficiently low to quantify the concentrations observed in water samples, which ranged from 18.73 to 71.00 mg/L for Ca^2+^, 1.88–5.77 mg/L for Mg^2+^, 3.53–10.58 mg/L for Na^+^, 0.50–4.28 mg/L for K^+^, 13.40-280.67 mg/L for HCO_2_^−^, 8.33-25.00 mg/L for Cl^−^, 2.14–24.50 mg/L for NO_2_^−^, 13.33-64.00 mg/L for SO_4_^2−^, 0.004–0.144 mg/L for Fe, 0.00025–0.00156 mg/L for Cd, 0.00056–0.0015 mg/L for Cr, 0.002–0.009 mg/L for Cu, 0.00005-0.00784 mg/L for Hg, 0.00167-0.042 mg/L for Mn, 0.002-0.00525 mg/L for Ni, 0.002 mg/L for Pb, 0.07175–0.16067 mg/L for Sr, and 0.0025–0.03938 mg/L for Zn.

A rigorous quality assurance and quality control (QA/QC) program was implemented throughout the analytical process to ensure data reliability. Daily instrument calibration was performed using multi-element standards (Inorganic Ventures, Christiansburg, VA, USA) with 5-point calibration curves (r^2^ > 0.9995). Method blanks were analyzed every 10 samples to check for contamination, and laboratory control samples (LCS) were analyzed every 20 samples to verify accuracy. Continuing calibration verification (CCV) standards were analyzed every 10 samples to ensure calibration stability throughout the analytical run. Internal standards (Sc, Y, In, Tb, Bi) were used to correct for matrix effects and instrument drift, particularly important for the varying matrix compositions encountered across different sampling locations and seasons.

The accuracy of analytical methods was assessed through spike recovery tests and analysis of certified reference materials. Spike recoveries were performed on 10% of samples, with recovery rates closely matching the concentration ranges observed in this study. For trace metals with moderate concentrations (Fe, Mn, Cu, Zn) ranging from 0.004 to 0.144 mg/L, 0.00167–0.042 mg/L, 0.002–0.009 mg/L, and 0.0025–0.03938 mg/L respectively, recovery rates were 90–110%. For ultra-trace metals (Cd, Pb, Hg) with very low concentrations (0.00025–0.00156 mg/L, 0.002 mg/L, and 0.00005-0.00784 mg/L respectively), recovery rates were 85–115%. Additionally, NIST SRM 1643f (Trace Elements in Water) was analyzed with recoveries of 92–108%, further validating our analytical methods.

Precision was evaluated through duplicate analyses performed on 10% of samples. The relative percent difference (RPD) for trace elements with lower concentrations (Fe, Mn, Cu, Zn, Cd, Cr, Pb, Hg) was kept below 20%. The relative standard deviation (RSD) for all elements was maintained below 5% for concentrations greater than 10 times the MDL, which applied to most of our measured parameters. For parameters near the detection limit, such as some Cd, Hg, and Pb measurements, the RSD was maintained below 15%, still within acceptable limits for environmental analysis.

Based on the principle of electrical neutrality, where the total number of cations equals the total number of anions in meq/L was employed to evaluate anion-cation balance errors. The CBE for all analyzed samples was within the acceptable range of ± 5%. The standard equation for balance was utilized (Eq. 1).

1 $${\text{CBE }}\; = \; - / + *100$$

The Maps were created using QGIS 3.42 as a free source software. Due to the large distance between the sampling points, the visualization and distribution of the concentration were represented in form of circles with different colors to avoid any over estimation between the sampling points.

### Canadian water quality index (CWQI)

The CWQI was established by the Canadian government in 2001. CWQI integrates three factors to compute the ultimate CWQI score^39,55,56^. The formulation of CWQI is outlined as follows.

F1 represents the percentage of parameters (out of the total parameters measured) that do not meet the criteria at least once during the specified period (Eq. 2)^39,57^.

2 $${\text{F}}1\; = \:100\: \times \:\frac{{{\text{Number}}\:{\text{of}}\:{\text{failed}}\:{\text{parameters}}}}{{{\text{Total}}\:{\text{number}}\:{\text{of}}\:{\text{parameters}}}}$$

F2 represents the proportion of tests that fail to satisfy typical requirements (Eq. 3) ^39,58^.

3 $${\text{F}}2 = \:100\: \times \:\frac{{{\text{Number}}\:{\text{of}}\:{\text{failed}}\:{\text{tests}}}}{{{\text{Total}}\:{\text{number}}\:{\text{of}}\:{\text{tests}}}}$$

F3 reflects how far test results depart from typical norms and is computed in three steps^39,58^.

(i) The term “excursion” refers to how often a concentration exceeds the specified limit. In situations where the measured value cannot exceed the threshold (Eq. 4):
  4 $${\text{Excursion}}_{{\text{i}}} \; = \;\left| {\frac{{{\text{Failed}}\:{\text{test}}\:{\text{value}}_{{\text{i}}} }}{{{\text{Objective}}_{{\text{i}}} }}} \right|\: - 1$$
  In situations where the measured value cannot fall below the threshold (Eq. 5):
  5 $${\text{Excursion}}_{{\text{i}}} \; = \:\left| {\frac{{{\text{Objective}}_{{\text{i}}} }}{{{\text{Failed}}\:{\text{test}}\:{\text{value}}_{{\text{i}}} }}} \right|\: - 1$$
(ii) “nse” reflects the overall deviation from standards, calculated as the sum of all excursions divided by total tests (meeting/failing) (Eq. 6):
  6 $${\text{nse}}\; = \:\frac{{\sum\nolimits_{{{\text{i}} = 1}}^{{\text{n}}} {{\text{excursion}}_{{\text{i}}} } }}{{{\text{Total}}\:{\text{number}}\:{\text{of}}\:{\text{tests}}}}$$
(iii) F3 is derived from “nse” via an asymptotic function, scaling the sum of excursion values (meeting/failing) to a 0–100 range (Eq. 7).
  7 $${\text{F}}3\; = \:\frac{{{\text{nse}}}}{{{\text{nse}}\: \times \:0.01\: + \:0.01}}$$

The last CCME-WQI value is determined as follows (Eq. 8):

8 $${\text{CWQI}}\; = \:\frac{{\sqrt {1^{2} } + \:{\text{F}}2^{2} \: + \:{\text{F}}3^{2} }}{{1.732}}\: - 100$$

Table 1 shows different ranges and classification of the CWQI for each range.

**Table 1:** Classification of water quality according to CCME-WQI method^39,58^.

Parameter	Water quality index	Class	Interpretation
CWQI	0–44	Poor	Water quality is almost always threatened or impaired; conditions usually depart from natural or desirable levels
	45–64	Marginal	Water quality is frequently threatened or impaired; conditions often depart from natural or desirable levels.
	65–79	Fair	Water quality is usually protected but occasionally threatened or impaired; conditions sometimes depart from natural or desirable levels.
	80–94	Good	Water quality is protected with only a minor degree of threat or impairment; conditions rarely depart from natural or desirable levels.
	95–100	Excellent	Water quality is protected with a virtual absence of threat or impairment, conditions very close to natural or pristine levels.

### Heavy metal pollution index (HPI) and Heavy Metal Index (HMI)

The HPI serves as an effective tool for quantifying the extent of HM pollution in water bodies^59^. It is particularly valuable in evaluating water suitability for consumption in the presence of heavy metals. The HPI is determined through attribute ratings and weighted mean calculations, where each pollutant is assigned, a weight based on its significance. A grading system, ranging from 0 to 1, emphasizes the relevance of specific water quality parameters in relation to established reference standards^60–62^. The calculations for HPI are outlined in the following equations (Eq. 9).

9 $${\text{HPI}} = \frac{{\sum\nolimits_{{{\text{i}} = 1}}^{{\text{n}}} {{\text{W}}_{{\text{i}}} {\text{Q}}_{{\text{i}}} } }}{{\sum\nolimits_{{{\text{i}} = 1}}^{{\text{n}}} {{\text{Wi}}} }}$$

Here, Q_i_ represents the sub-index for each parameter, while n refers to the total number of parameters analyzed. w_i_ denotes the weight assigned to each parameter, calculated as 1/S_i_, where S_i_ is the WHO 2017 standard value^63^ of the respective parameter. The sub-index boundary, Q_i_ is determined using Eq. 10.

10 $${\text{Q}}_{{\text{i}}} = \sum\nolimits_{{{\text{i}} = 1}}^{{\text{n}}} {100\: \times \:\frac{{C_{{\text{i}}} }}{{S_{{\text{i}}} }}}$$

The HPI indicator measures the concentration levels of HMs. This index is commonly evaluated on an adapted five-tier scale: Excellent quality (HPI below 15), Good to Intermediate quality (HPI between 15 and 30), Poor to Unsuitable quality (HPI above 30), Very Poor quality (HPI between 76 and 100), and Unsuitable (HPI above 100)^64,65^.

The HMI is a valuable metric for evaluating the cumulative impact of potentially toxic elements (PTEs) on human health, thus providing an assessment of potable water quality. This index operates on the principle that toxicity increases proportionally with the concentration of PTEs. Exposure to these metals poses various toxicological risks, both immediate and long-term, affecting multiple organs. Calculating the HMI involves a detailed assessment of current metal concentrations; if levels exceed their Higher Allowable Limits (HAL), it signals a decline in groundwater quality^60,62^. The HMI, introduced by Tamasai and Cini, is defined in Eq. (11).

11 $${\text{HMI}} = \sum\nolimits_{{{\text{i}} = 1}}^{{\text{i}}} {\frac{{{\text{C}}_{{\text{i}}} }}{{{\text{HAL}}_{{\text{i}}} }}}$$

In this context, $\:{\text{C}}_{\text{i}}$ represents the concentration of each heavy metal being analyzed, while $\:\text{H}\text{A}\text{L}$ refers to the higher allowable limits for each metal $\:\text{i}$. According to Withanachchi^66^ the HMI is categorized into six levels to indicate contamination severity: Very Clean (HMI < 0.3), Clean (0.3 < HMI < 1), Somewhat Polluted (1 < HMI < 2), Moderately Polluted (2 < HMI < 4), Highly Polluted (4 < HMI < 6), and Seriously Polluted (HMI > 6). These categories highlight the potential health risks associated with PTE contamination, providing a framework for assessing the quality of water resources and their potential health impacts.

### The potential ecological risk index (ERI)

First proposed by Hakanson in 1980 ^67^, the Potential Ecological Risk Index (ERI) offers a systematic approach to evaluate the environmental risks posed by heavy metals in a specific ecosystem. This index takes into account factors such as heavy metal concentrations, their toxicity, environmental sensitivity, types, and baseline background levels^68^. Although widely utilized across various scientific fields, our study focuses on applying this index to assess the ecological risks of heavy metals and strontium (Sr) in springs water. The ERI is calculated using the following formula (Eq. 12):

12 $$\:\text{E}\text{R}\text{I}=\sum\:{\text{E}}_{\text{r}}^{\text{i}}={\text{T}}_{\text{r}}^{\text{i}}\times\:\left\{\frac{{\text{C}}_{\text{c}\text{o}\text{n}}^{\text{i}}}{{\text{C}}_{\text{b}\text{g}}^{\text{i}}}\right\}$$

Here, E_r_ denotes the ecological risk factor of a given substance, while T_r_ is the toxic response factor associated with each specific heavy metal (Table 2). $\:{\text{C}}_{\text{c}\text{o}\text{n}}^{\text{i}}$ represents the concentration of each heavy metal in the sample, and $\:{\text{C}}_{\text{b}\text{g}}^{\text{i}}\:\:$indicates the background levels of these metals (Table 2). The Potential Ecological Risk Index (ERI) provides a comprehensive metric for assessing the total ecological risk from contamination. ERI values fall into four risk categories: low (under 30), moderate (30–60), considerable (60–120), and very high (above 120)^60,64^.

### Health risk assessment

The potential health risks to inhabitants from using the water were assessed through an evaluation using Eqs. 13, 14.

13 $$\:{\text{C}\text{D}\text{I}}_{\text{o}\text{r}\text{a}\text{l}}=\frac{\text{E}\text{F}\:\times\:\text{I}\text{R}\times\:\text{E}\text{D}}{\text{B}\text{W}\times\:{\text{A}\text{T}}_{\text{n}\text{c}}}\:\times\:{\text{C}}_{\text{c}\text{o}\text{n}}$$

14 $$\:{\text{C}\text{D}\text{I}}_{\text{d}\text{e}\text{r}\text{m}\text{a}\text{l}}=\frac{\text{E}\text{F}\:\times\:\text{E}\text{T}\times\:\text{E}\text{D}\times\:\text{K}\text{p}\times\:\text{S}\text{A}\times\:\text{C}\text{F}}{\text{B}\text{W}\times\:{\text{A}\text{T}}_{\text{n}\text{c}}}\:\times\:{\text{C}}_{\text{c}\text{o}\text{n}}$$

Here, CDI represents the chronic intake dose through daily ingestion and dermal exposure (mg/kg/day). Table 2 provides the corresponding risk values. Non-cancer risks were evaluated by calculating the hazard quotient (HQ)^69,70^ and hazard index (HI) for both ingestion and dermal contact using Eqs. 15, 16, 17. An HQ or HI value below 1 indicates no significant risk, whereas values above 1 suggest potential health risks^62,71,72^.

**Table 2:** The utilized parameters in the current risk investigation.

Parameters	Symbol	Unit	Adult	Child	Reference
Metals concentration	C	ppm			–
Ingestion rate	IR	L/day	2.2	1.8	^60,61^
Exposure frequency	EF	days/year	350	350	^60,61^
Average time (carcinogenic)	AT	days	25,550	25,550	^60,61^
Average time (non-carcinogenic)	AT	days	10,950	2190	^69,70^
Exposure duration	ED	years	70	6	^60,61^
Body weight	BW	kg	70	15	^60,61^
Surface area	SA	cm^2^	18,000	6600	^60,61^
Conversion factor	CA	L cm^− 3^	1 E−3	1 E−3	
Permeability coefficient	K_p_	cm/h			
Exposure time	ET	h/day	0.58	1	^60,61^

15 $$\:{\text{H}\text{Q}}_{\text{d}\text{e}\text{r}\text{m}\text{a}\text{l}}=\frac{{\text{C}\text{D}\text{I}}_{\text{d}\text{e}\text{r}\text{m}\text{a}\text{l}}}{{\text{R}\text{f}\text{D}}_{\text{d}\text{e}\text{r}\text{m}\text{a}\text{l}}}\:\:\:,\:{\text{H}\text{Q}}_{\text{o}\text{r}\text{a}\text{l}}=\frac{{\text{C}\text{D}\text{I}}_{\text{o}\text{r}\text{a}\text{l}}}{{\text{R}\text{f}\text{D}}_{\text{o}\text{r}\text{a}\text{l}}}$$

16 $$\:{\text{R}\text{e}\text{f}\text{e}\text{r}\text{e}\text{n}\text{c}\text{e}\:\text{d}\text{e}\text{r}\text{m}\text{a}\text{l}\:\text{d}\text{o}\text{s}\text{e}\text{s}\:(\text{R}\text{f}\text{D}}_{\text{d}\text{e}\text{r}\text{m}\text{a}\text{l}})={\text{R}\text{f}\text{D}}_{\text{o}\text{r}\text{a}\text{l}}\times\:\text{A}\text{B}\text{S}$$

In the conclusive phase, the comprehensive non-carcinogenic risks were evaluated by computing the hazard index (HI) according to Eq. 17^73^.

17 $$\:\text{H}\text{I}={\text{H}\text{Q}}_{\text{o}\text{r}\text{a}\text{l}}+{\text{H}\text{Q}}_{\text{d}\text{e}\text{r}\text{m}\text{a}\text{l}}$$

Toxic metals exhibiting a hazard quotient (HQ) or hazard index (HI) surpassing 1 are indicative of potential deleterious effects on human health, whereas those with an HI or HQ below 1 are regarded as unlikely to cause adverse consequences^60,61,71^.

### Carcinogenic human health risk method

Using the approach outlined by Li and Zhang^74^ the carcinogenic risk (CR) was calculated using Eq. 18 and Table 3. This value represents the lifetime probability of developing cancer from exposure to carcinogenic substances. Typically, the acceptable risk range falls between 1 E−4–1 E−6 ^74^

18 $$\:\text{C}\text{R}=\text{C}\text{D}\text{I}\:\times\:\:\text{C}\text{S}\text{F}$$

**Table 3:** The parameters for the computation of HQ, HI, RI and CR.

Heavy metal (HM)	Cd	Cr	Cu	Fe	Hg	Mn	Ni	Pb	Sr	Zn	Ref
Oral reference dose. RfD oral (mg/kg/day)	0.0005	0.003	0.04	0.7	0.0003	0.024	0.02	0.0014	0.6	0.3	^62,75^
Dermal reference dose. Rfd Dermal (mg/kg/day)	0.000025	0.000075	0.012	0.14	0.000021	0.00096	0.0008	0.00042	0.12	0.06	^62,75^
Dermal absorption fraction. ABS	0.05	0.025	0.3	0.2	0.07	0.04	0.04	0.3	0.2	0.2	^62,75^
Permeability Coefficient. Kp	0.001	0.002	0.001	0.001	0.001	0.001	0.0002	0.0001	0.001	0.0006	^62,75^
Oral cancer slope factor. CSFing mg/kg/day	6.1	0.5						0.5			^62,75^
Dermal cancer slope factor. CSFder	6100	500						500			^62,75^
Toxic response factor. Tr values	30	2	5	1	40	1	5	5	1	1	^67^
Background value ppm. C_bg_	0.2	35	55	15,000	0.8	600	20	12.5	350	70	^76^

### Monte Carlo simulation (MCS)

The MCS in this study aimed to assess the probability distributions of factors like HM concentration, ET, ED, AT, EF, Kp, BW and SA. By integrating this method with the USEPA health risk assessment, the model evaluated uncertainties in cancer risk (CR) and HQ values for both children and adults. The analysis used 10,000 iterations in Python, producing similar observed and predicted HQ values, confirming the model’s accuracy^60,61,77^.

### Method for evaluating industrial water quality

In recent times, industrialization has been recognized as a crucial factor in driving significant economic progress and growth, especially in industrial countries. This implies that the need for high-quality water in various industries and industrial processes is expected to rise steadily. As a result, any nation striving to achieve industrialization goals must prioritize ensuring a reliable supply of quality water for its industrial sector. However, providing an adequate water supply often presents a considerable challenge for this sector^78^. This issue is similarly affecting many households. In fact, a large number of homes, businesses, and institutions are currently facing various negative impacts related to water corrosion and scaling^78–80^. The approach used in this study to assess commercial water quality involved applying several indices, such as CSMR, RI, PSI, RSI, LSI, AI, and LI, to analyze the water’s corrosivity and scaling potential. The formulas for calculating the corrosion and scaling potentials (CSPs) of these water sources (Eqs. 19–31) are provided below, and the CSP index standards are listed in Table S1.

19 $$\:\text{R}\text{e}\text{v}\text{e}\text{l}\text{l}\text{e}\:\text{i}\text{n}\text{d}\text{e}\text{x}\:\left(\text{R}\text{I}\right)=\:\frac{{\text{C}\text{l}}^{-}}{{\text{H}\text{C}\text{O}}_{3}^{-}}$$

20 $$\:\text{C}\text{h}\text{l}\text{o}\text{r}\text{i}\text{d}\text{e}-\text{s}\text{u}\text{l}\text{p}\text{h}\text{a}\text{t}\text{e}\:\text{m}\text{a}\text{s}\text{s}\:\text{r}\text{a}\text{t}\text{i}\text{o}\:\left(\text{C}\text{S}\text{M}\text{R}\right)=\:\frac{{\text{C}\text{l}}^{-}}{{\text{S}\text{O}}_{4}^{2-}}$$

21 $$\:\text{L}\text{a}\text{n}\text{g}\text{e}\text{l}\text{i}\text{e}\text{r}\:\text{S}\text{a}\text{t}\text{u}\text{r}\text{a}\text{t}\text{i}\text{o}\text{n}\:\text{I}\text{n}\text{d}\text{e}\text{x}\:\left(\text{L}\text{S}\text{I}\right)=\text{p}\text{H}-\:\text{p}\text{H}\text{s}$$

22 $$\:\text{A}=\:\frac{{\text{l}\text{o}\text{g}}_{10}\left(\text{T}\text{D}\text{S}\right)-1}{10}$$

23 $$\:\text{B}=\:-13.12\times\:{\text{L}\text{o}\text{g}}_{10}\:\left(\text{T}\:\text{k}\text{e}\text{l}\text{v}\text{i}\text{n}\right)+34.55$$

24 $$\:\text{C}=\:{\text{L}\text{o}\text{g}}_{10}\:\left({\text{C}\text{a}}^{2+}\right)-0.4$$

25 $$\:\text{D}=\:{\text{L}\text{o}\text{g}}_{10}\left(\text{A}\text{l}\text{k}\right)$$

26 $${\text{pHs = (9}}{\text{.3 + A + B)}}$$

27 $${\text{Larson}} - {\text{Skold}}\:{\text{index}}\left( {{\text{LS}}} \right)\; = \:\frac{{{\text{Cl}}^{ - } + \:{\text{SO}}_{4}^{{2 - }} }}{{{\text{HCO}}_{3}^{ - } }}$$

28 $$\:\text{A}\text{g}\text{g}\text{r}\text{e}\text{s}\text{s}\text{i}\text{v}\text{e}\:\text{i}\text{n}\text{d}\text{e}\text{x}\:\left(\text{A}\text{I}\right)=\text{p}\text{H}+\:{\text{L}\text{o}\text{g}}_{10}(\text{A}\text{l}\text{k}\text{a}\text{l}\text{i}\text{n}\text{i}\text{t}\text{y}\times\:\text{H}\text{a}\text{r}\text{d}\text{n}\text{e}\text{s}\text{s}\:(\text{a}\text{s}\:{\text{C}\text{a}\text{C}\text{O}}_{3}\left)\:\right)$$

29 $$\:\text{R}\text{y}\text{z}\text{n}\text{a}\text{r}\:\text{s}\text{t}\text{a}\text{b}\text{i}\text{l}\text{i}\text{t}\text{y}\:\text{i}\text{n}\text{d}\text{e}\text{x}\:\left(\text{R}\text{S}\text{I}\right)=2\:\text{p}\text{H}\text{s}-\text{p}\text{H}$$

30 $$\:\text{P}\text{u}\text{c}\text{k}\text{o}\text{r}\text{i}\text{u}\text{s}\:\text{s}\text{c}\text{a}\text{l}\text{i}\text{n}\text{g}\:\text{i}\text{n}\text{d}\text{e}\text{x}\:\left(\text{P}\text{S}\text{I}\right)=2\:\text{p}\text{H}\text{s}-\text{p}\text{H}\text{e}\text{q}$$

31 $$\:\text{p}\text{H}\text{e}\text{q}=1.465\times\:\:{\text{L}\text{o}\text{g}}_{10}\left(\text{A}\text{l}\text{k}\text{a}\text{l}\text{i}\text{n}\text{i}\text{t}\text{y}\right)+4.54$$

## Results and discussion

**Fig. 2:**
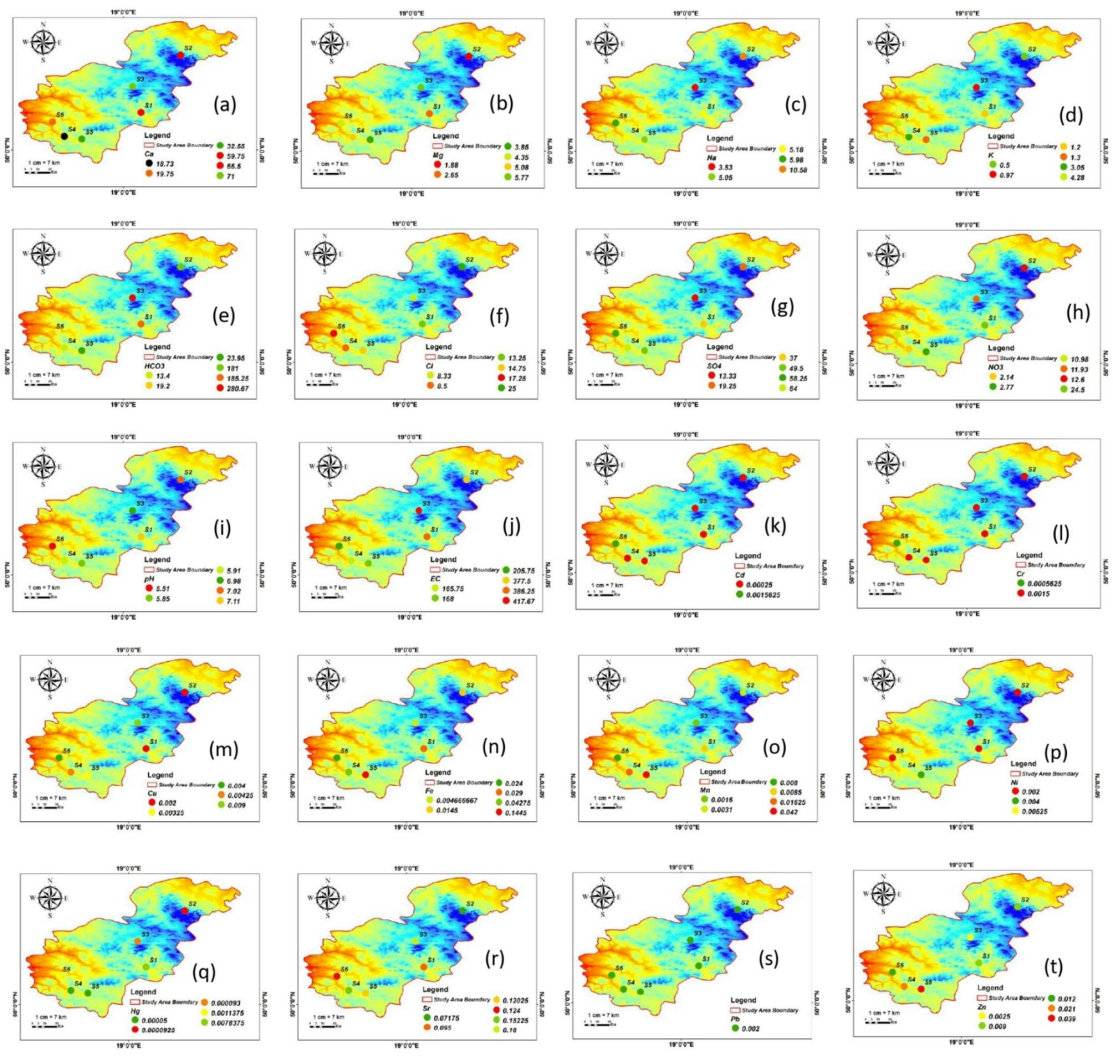
Spatial distribution of (**a**) Ca^2+^; (**b**) Mg^2+^; (**c**) Na^+^; (**d**) K^+^; (**e**) HCO_3_^-^; (**f**) NO_3_^−^; (**g**) SO_4_^2-^; (**h**) Cl^-^; (**i**) pH; (**j**) EC; (**k**) Cd; (**l**) Cr; (**m**) Cu; (**n**) Fe; (**o**) Hg; (**p**) Mn; (**q**) Ni; (**r**) Pb; (**s**) Sr; and (**t**) Zn.

All the measured parameters in each season were reported in Table 1. These fluctuations reveal both regional and temporal differences in water quality, highlighting how local environmental factors, such as weather patterns, hydrological cycles, and land-use practices, interact with seasonal shifts in water chemistry. Figure 2 shows the concentration of the measured parameters in form of average value to differentiate between each location characteristics.

### Seasonal patterns in major ions

The seasonal variations in major ions across all sampling locations generally follow consistent patterns influenced by rainfall, evaporation, temperature changes, and mineral weathering processes. Typically, concentrations of Ca^2+^, Mg^2+^, and HCO_2_^−^ peak during colder months and decline in warmer periods, while Na^+^ and Cl^−^ often show summer increases due to evaporative concentration. These patterns vary in magnitude across locations, reflecting differences in local geology, groundwater contribution, and potential anthropogenic inputs.

The seasonal variations in Ca^2+^ concentrations across the six sampling locations demonstrate a consistent trend of high concentrations in colder months and substantial declines in warmer periods. At Leśniów, Ca^2+^ levels show a marked decrease from a winter high of 90 mg/L in November and February to a late-summer low of 19 mg/L in August. This significant seasonal drop reflects dilution or precipitation effects, likely influenced by higher rainfall and warmer temperatures. Zygmunt and Halszka follow similar seasonal fluctuations; in Zygmunt, Ca^2+^ levels peak at 81 mg/L in November and February, drop to 44 mg/L in May, and partially recover in August at 60 mg/L. This recovery, though less pronounced than at Leśniów, still highlights the seasonal dynamics of rainfall and evaporation. Halszka experiences a slightly different cycle, with Ca^2+^ levels peaking at 96 mg/L in November, dropping to 45 mg/L in February, and rising again to 72 mg/L in May. Dobro Woda and Święto Woda exhibit more moderate yet clear seasonal variations, with Ca^2+^ peaking around 27 mg/L in colder months and reaching lower levels in late summer (70 mg/L in Święto Woda). Zimny Sztok displays more stable seasonal variation, with Ca^2+^ concentrations fluctuating between 24 mg/L in November and a winter peak of 32 mg/L in February, followed by a decline during the summer months, indicating seasonal groundwater contributions. This pattern indicates that seasonal fluctuations in Ca^2+^ concentrations are predominantly influenced by rainfall, evaporation, and groundwater influx, with consistent trends across most locations.

Mg^2+^ concentrations across the six locations show notable winter peaks, driven by geochemical processes like mineral dissolution, which become more active in colder months, and drop as temperatures rise. In Leśniów, Mg^2+^ levels are lowest in August at 5 mg/L, with levels reaching 5.1 mg/L during winter. Zygmunt mirrors this pattern with Mg^2+^ concentrations starting at 0.73 mg/L in November, peaking at 2.6 mg/L by February, and remaining relatively stable throughout the summer. Halszka’s Mg^2+^ levels vary slightly more, peaking at 6.7 mg/L in November and declining to 3.6 mg/L in February. In Dobro Woda, Mg^2+^ displays a similar winter increase, reaching its highest in February at 7.2 mg/L before dropping to 3 mg/L in May. Święto Woda and Zimny Sztok follow this general seasonal trend; for instance, Święto Woda’s Mg^2+^ peaks at 5.4 mg/L in February and declines through the warmer months, while Zimny Sztok’s Mg^2+^ concentrations show consistent increases, peaking in February at 6.8 mg/L, possibly due to enhanced mineral dissolution during this period. The observed fluctuations demonstrate the combined influence of groundwater input and mineral weathering in colder months, particularly across locations where groundwater has a pronounced impact on water chemistry.

The elevated concentrations of Ca^2+^ and Mg^2+^ during winter months can be attributed to several interconnected hydrogeochemical processes. During winter, reduced temperatures slow down biological activity and decrease evapotranspiration rates, leading to longer residence times of groundwater in contact with carbonate minerals^81,82^. This extended contact time enhances the dissolution of calcite (CaCO_3_) and dolomite (CaMg(CO_3_)_2_) according to the following reactions:

$${\text{CaCO}}_{{\text{3}}} + {\text{ H}}_{{\text{2}}} {\text{CO}}_{{\text{3}}} \to {\text{ Ca}}^{{{\text{2}} + }} + {\text{ 2HCO}}_{{\text{3}}} ^{ - }$$

$${\text{CaMg }}\left( {{\text{CO}}_{{\text{3}}} } \right)_{{\text{2}}} + {\text{ 2H}}_{{\text{2}}} {\text{CO}}_{{\text{3}}} \to {\text{ Ca}}^{{{\text{2}} + }} + {\text{ Mg}}^{{{\text{2}} + }} + {\text{ 4HCO}}_{{\text{3}}} ^{ - }$$

Additionally, winter precipitation often has lower pH due to increased CO_2_ solubility at lower temperatures, creating more aggressive water that enhances carbonate mineral dissolution^83,84^. The reduced biological uptake of these cations during dormant winter periods also contributes to their accumulation in groundwater^85^.

K^+^ levels display less dramatic seasonal fluctuations, yet still reflect distinct patterns tied to dilution and mineral dissolution. In Leśniów, K^+^ peaks at 1.5 mg/L in February, dropping to a low of 0.5 mg/L by May and raised again in August to 2.3 mg/L. Zygmunt exhibits a winter balanced of 0.5 mg/L, while Halszka shows a smaller range, peaking at 1.3 mg/L in May and declining to 1.1 mg/L by November. Dobro Woda and Święto Woda follow similar trends with high winter K^+^ levels around 2.5 mg/L, rising to nearly threefold that concentration in August. In Zimny Sztok, K^+^ levels display a gradual decline from a high of 6 mg/L in November to 2.4 mg/L by August. This seasonal dynamic reveals a likely reduction of K-rich mineral leaching during the warmer months, as rainfall dilutes surface water.

HCO_2_^−^ concentrations peak in winter and decline in the summer across all locations, likely due to temperature-induced changes in carbon dioxide solubility and biological activity. At Leśniów, HCO_2_^−^ levels reach 208 mg/L in November and decrease to 152 mg/L in August. Zygmunt also follows this pattern, with HCO_2_^−^ peaking at 201 mg/L in February and gradually decreasing through summer. Halszka’s HCO_2_^−^ shows a notable high in winter at 307 mg/L, dropping to 252 mg/L by May. Similarly, Dobro Woda exhibits seasonal high HCO_2_^−^ concentrations in winter (6 mg/L in February) and lower levels in August (29 mg/L). Święto Woda and Zimny Sztok reflect this same pattern; in Święto Woda, HCO_2_^−^ peaks at 29.6 mg/L in November and drops to 22.1 mg/L in August, while in Zimny Sztok, levels decrease from a high of 34 mg/L in November to a late-summer low of 24 mg/L. The pattern indicates that HCO_2_^−^ concentrations are strongly influenced by the seasonal availability of dissolved CO₂, with temperature and biological activity affecting its solubility.

NO_2_^−^ levels fluctuate seasonally, with winter and early spring peaks at most sites, which may reflect agricultural runoff or atmospheric deposition. For instance, at Leśniów, NO_2_^−^ concentrations decrease slightly in May (21 mg/L) and increase again in August (27 mg/L), likely influenced by agricultural practices. Zygmunt’s NO_2_^−^ levels are generally stable but show a slight winter increase, while Halszka and Dobro Woda exhibit early spring peaks, likely due to runoff from fertilized fields. In Zimny Sztok, NO_2_^−^ levels reach 11 mg/L in February and drop to 7.9 mg/L in May, with a slight rise again in August (12 mg/L), further emphasizing the potential impact of agricultural runoff.

Cl^−^ concentrations show strong seasonality, especially in summer, possibly from evaporative concentration and runoff inputs. In Leśniów, Cl^−^ levels rise from a winter low of 13 mg/L in February to a high of 14 mg/L in August. Zygmunt follows this seasonal trend, with Cl^−^ levels decreasing from 28 mg/L in February to 24 mg/L in August, mirroring Na’s seasonal peaks. Halszka, Dobro Woda, and Święto Woda also exhibit distinct Cl^−^ peaks in summer months, likely from evaporative processes that concentrate Cl^−^ and Na^+^ ions. In Zimny Sztok, Cl^−^ levels peak in August at 17 mg/L after lower levels in colder months. The observed fluctuations indicate a significant influence of seasonal temperature and rainfall cycles on Cl^−^ concentrations, with increased evaporation in warmer months concentrating Cl^−^.

The increased concentrations of Na^+^ and Cl^−^ during summer months result from multiple factors. Elevated summer temperatures increase evaporation rates, leading to concentration of dissolved salts in the remaining water^34,86^. This evaporative concentration effect is particularly pronounced for conservative ions like Cl^−^ that do not participate in mineral precipitation reactions under typical groundwater conditions^87^. Furthermore, summer months often coincide with increased human activities in recreational areas where some of sampling sites are located. Road salt application during winter months can create a delayed release of Na^+^ and Cl^−^ into groundwater systems, with peak concentrations often observed during subsequent summer months due to seasonal flushing patterns^88,89^. Agricultural activities, including fertilizer application, also contribute to elevated Na^+^ and Cl^−^ concentrations during growing seasons^90^. The anthropogenic influence is particularly relevant for springs located near recreational areas (Dobro Woda, Święto Woda, and Zimny Sztok), where increased summer tourism and associated activities may contribute to localized contamination sources^91^.

The study area experiences a temperate continental climate with distinct seasonal variations that significantly influence hydrogeochemical processes. Mean annual temperature ranges from 7 to 8 °C, with winter temperatures often dropping below freezing (December-February average: −2 to 2 °C) and summer temperatures reaching 18–20 °C (June-August average). This temperature range creates optimal conditions for enhanced carbonate mineral dissolution during colder months when CO_2_ solubility increases and biological activity decreases. Annual precipitation varies between 700 and 750 mm, with maximum precipitation occurring during summer months (July–August: 80–100 mm/month) and minimum during winter (January–February: 30–40 mm/month). This precipitation pattern, combined with seasonal evapotranspiration rates (ranging from 100 mm/month in summer), creates distinct hydrological conditions that influence groundwater recharge and chemical evolution. Relative humidity reaches maximum values of 85% during winter months, contributing to reduced evaporation rates and longer water-rock interaction times. Conversely, summer humidity drops to approximately 70%, coinciding with increased evaporation rates that concentrate dissolved constituents. These climatic conditions create a seasonal cycle where winter months favor enhanced mineral dissolution due to lower temperatures, higher CO_2_ solubility, and reduced biological activity, while summer months promote evaporative concentration of conservative ions and increased anthropogenic inputs from recreational activities.

Na^+^ levels display distinct summer increases in most locations, likely a result of evaporation and reduced rainfall, which concentrate dissolved ions. In Leśniów, Na^+^ levels rise from a low of 2.5 mg/L in May to 6.9 mg/L by August. Zygmunt follows a similar seasonal pattern, with Na^+^ peaking in February at 13 mg/L, decreasing in May, and rising again in August to 11 mg/L. Halszka, Dobro Woda, and Święto Woda experience moderate summer increases as well; for instance, Dobro Woda’s Na^+^ peaks at 8.4 mg/L in August, up from 2.9 mg/L in May, possibly due to increased groundwater inflow or mineral dissolution during warmer months. Zimny Sztok also shows minor Na^+^ fluctuations, with levels rising from 7.1 mg/L in November to 9 mg/L in February and it was 5 mg/L in August. The data illustrate a common pattern of ion concentration increase due to summer evaporation and limited freshwater inflow.

The concentration of SO_4_^2−^ In Leśniów, sulfate concentrations peak in the winter months, reaching up to 40 mg/L in February, likely due to reduced dilution from lower precipitation levels and potential leaching of SO_4_^2−^ minerals from nearby soils or bedrock. During the summer months, SO_4_^2−^ levels decrease to around 38 mg/L by August, possibly from increased rainfall, which dilutes sulfate concentrations. Additionally, biological uptake during warmer months might contribute to this seasonal decline in SO_4_^2−^ levels, as plant and microbial activity increases. At Zygmunt, a similar seasonal pattern is observed, with SO_4_^2−^ concentrations rising in the colder months to approximately 55 mg/L in February, then gradually dropping to around 28 mg/L in August. This pattern suggests a decrease in sulfate solubility and a dilution effect during the warmer, wetter summer months. The influence of local runoff could play a role here as well, as agricultural runoff often contains sulfur-based compounds, contributing to the higher winter levels that subsequently decrease in summer when dilution and plant uptake are most active. Halszka follows a comparable trend, with SO_4_^2−^ concentrations peaking at about 23 mg/L in February and declining to around 16 mg/L in May before stabilizing at 22 mg/L in August. This location-specific trend might reflect the combined effects of reduced dilution in the colder months and possible anthropogenic inputs. The relatively high SO_4_^2−^ levels suggest that groundwater or human activities, such as agriculture or industry, may be contributing to the elevated winter concentrations, which then decrease as seasonal precipitation increases during the summer. In Dobro Woda and Święto Woda, SO_4_^2−^ concentrations reach similar winter peaks, around 70 mg/L, which then decline to nearly quarter, around 50 mg/L, by August. This pattern indicates strong seasonal influences, with increased SO_4_^2−^ in colder months likely stemming from decreased dilution effects and higher levels of runoff from nearby land, potentially containing residual SO_4_^2−^ from fertilizers. As rainfall and temperature increase through the summer, the dilution effect becomes more pronounced, reducing SO_4_^2−^ levels. Finally, Zimny Sztok displays a less pronounced but consistent seasonal trend, with SO_4_^2−^ concentrations reaching around 57 mg/L in November and dropping to approximately 55 mg/L by August. This relatively muted fluctuation might indicate that Zimny Sztok experiences lower anthropogenic influences than other sites, with seasonal SO_4_^2−^ levels more directly governed by natural processes like precipitation and mineral dissolution rather than human activity.

pH levels across these sites generally remain within a slightly alkaline range, though with subtle shifts tied to seasonal and local influences. During the colder months, pH levels tend to be lower, particularly in Leśniów, where readings range from 7 to 7.3 in November and February. This decrease may be associated with reduced biological activity and possible runoff carrying acidic inputs or leachates from surrounding soils. In contrast, summer months exhibit a rise in pH, peaking around 7.05 in areas like Leśniów and Święto Woda. This seasonal increase is likely due to heightened photosynthetic activity by aquatic vegetation, which consumes dissolved CO_2_, thereby increasing pH levels slightly in the warmer period. Similarly, Zygmunt and Dobro Woda show comparable fluctuations, with winter readings near 7.13 and summer values close to 7.03, suggesting that seasonal biological and geochemical processes are relatively consistent across these locations.

EC, an indicator of total dissolved ions, also exhibits notable seasonal variation, influenced by factors such as evaporation, precipitation, and ion inputs from natural and anthropogenic sources. In general, EC levels tend to peak during the summer months, with the highest readings observed in August, particularly in Halszka and Leśniów, where EC measures reach up to 430 µS/cm and 467µS/cm, respectively. This increase is likely driven by enhanced evaporation rates during warmer months, which concentrate dissolved ions, alongside possible mineral dissolution and soil leaching intensified by seasonal hydrological changes. Conversely, the lowest EC values are recorded in spring, especially in May, as observed in Dobro Woda (139 µS/cm) and Święto Woda (149 µS/cm), likely reflecting dilution effects from increased rainfall and runoff. Zygmunt and Halszka follow a similar pattern, with EC peaking around February and declining through spring, corresponding to reduced ionic concentration due to dilution from precipitation. The seasonal EC variations observed across all six sites underscore the complex interplay of local weather patterns, geochemical interactions, and potential human influences that affect the concentration and mobility of dissolved ions in these water bodies. The seasonal variations in physicochemical parameters and HM amounts observed across the six sampling locations, Leśniów, Zygmunt, Halszka, Dobro Woda, Święto Woda, and Zimny Sztok, provide valuable insights into the dynamic interplay between natural geochemical processes and potential anthropogenic influences.

### Heavy metal concentrations

Heavy metals, including Cd, Cr, Cu, Ni, and Pb, remain relatively stable across seasons at all locations, indicating a steady background presence likely from local geology or low-level industrial contributions. However, certain metals display notable seasonal peaks. Fe levels, for instance, show a summer spike at several sites, suggesting enhanced mobilization from sediments or increased biological activity. At Leśniów, Fe levels remain low from November to May (0.002 mg/L) before rising to 0.11 mg/L in August. Zygmunt and Dobro Woda experience similar patterns, with Fe levels peaking in late summer. Hg also displays some seasonality, particularly at Leśniów, peaking in November (0.031 mg/L) before dropping during the following months. Sr concentrations exhibit peaks in August at most sites, suggesting increased mineral dissolution or groundwater influence during warmer months. Mn shows a consistent seasonal increase across locations, peaking in August, likely due to reducing conditions or enhanced leaching from surrounding soil.

### Hydrochemical investigation and water evolution

Hydrochemical facies analysis was selected as a primary methodological approach in this study due to its proven effectiveness in characterizing groundwater systems and identifying the dominant geochemical processes. This approach is particularly valuable for our study area in southern Poland for several key reasons: The use of Piper diagrams provides a standardized and internationally recognized method for classifying water types based on major ion compositions. The combination of Piper, Chadha, and Gibbs diagrams offers complementary insights that a single diagram approach cannot provide. While Piper diagrams effectively classify water types, Chadha diagrams specifically highlight ion exchange processes, and Gibbs plots distinguish between precipitation dominance, rock weathering, and evaporation-crystallization processes. The ionic ratios analysis complements the graphical methods by quantitatively assessing specific geochemical processes like silicate weathering, carbonate dissolution, and ion exchange. This multi-method approach provides robust evidence for the water-rock interaction mechanisms that would be impossible to determine through basic water quality parameters alone^9,26–28^. The hydrochemical facies approach, particularly through ionic ratios (NO_2_^2−^/Na^+^, SO_4_^2−^/Na^+^, Cl^−^/Na^+^), enables differentiation between natural geogenic processes and anthropogenic influences, which is critical for identifying pollution sources in the agriculturally and industrially active southern Poland region.

**Fig. 3:**
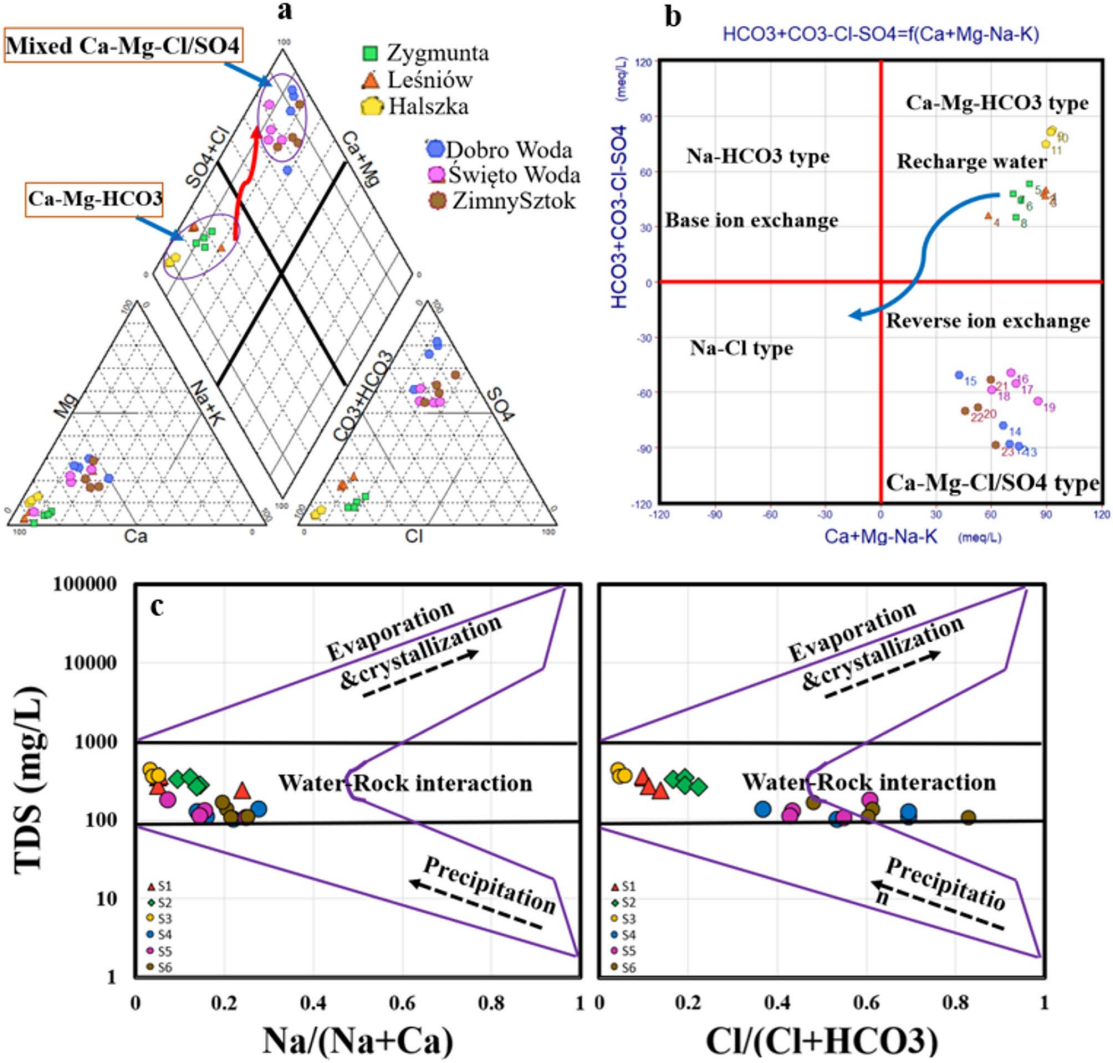
Graphical representation of the hydrochemical facies and the mechanism controlling the water chemistry: (**a**) Piper, (**b**) Chadha, and (**c**) Gibbs plot.

In the Piper diagram’s diamond shape, the water samples were divided into two main facies separated the six springs into two different groups (Fig. 3a). The first group includes three similar springs (Zygmunt, Leśniów, Halszka) dominated by Ca–Mg–HCO_3_ chemical facies and the remaining three springs located in Dobro Woda, Święto Woda, and ZimnySztok are dominated by mixed Ca–Mg-Cl/SO_4_ facies) with permanent hardness. The difference between the two groups of the springs revealed that the mechanism controlling the water chemistry are different due to natural and/or anthropogenic reactions. The group of springs located in Dobro Woda, Święto Woda, and Zimny Sztok revealed that the salinity factors (SO_4_^2−^ +Cl^−^) is greater than the alkalinity factors (HCO_3_^−^ + CO_3_^2−^) and the alkalis (Na^+^ +K^+^) are lower than the alkaline earths (Ca^2+^ + Mg^2+^) in all springs of the two groups. In contrast the remaining springs located in Zygmunt, Leśniów, Halszka showed the opposite characteristics with salinity indicators (SO_4_^2−^ + Cl^−^) is lower than the alkalinity factors (HCO_3_^−^ + CO_3_^2−^) which indicate that these regions receive relatively high annual recharge compared to the other locations.

Chadha diagram (Fig. 3b) which is advanced form of Piper diagram could confirm the findings of Piper plot as well the mechanism behind the change in the water type between the two groups of samples. Chadha diagram divided the springs samples where the first group (Zygmunt, Leśniów, and Halszka springs) representing the recharge water and dominated by Ca–Mg–HCO_3_ water type. The second group of samples located in Dobro Woda, Święto Woda, and Zimny Sztok were influenced by reverse Ca/Na ion exchange mechanism and dominated by mixed Ca–Mg–Cl/SO_4_ facies. There are several processes specifically the ion ratios could explain the evolution of the water chemistry that couldn’t be investigated through Piper and Chadha diagram. The chemistry evolution with the groundwater flow direction where the recharge area was dominated by Ca–Mg–HCO_3_ followed by water-rock interaction, carbonate, and silicate weathering and anthropogenic inputs giving signature of Ca–Mg–Cl/SO_4_ water type. The process controlling the presence of each component can be confirmed by various ionic ratios.

The Gibbs scatter plot (Fig. 3c) is often used to analyze the factors influencing water chemistry, dividing the graph into three main regions (Fig. 3a). The first region highlights the dominance of atmospheric precipitation, showing very low salinity and elevated ratios of Cl^−^/(Cl^−^ + HCO_3_^−^) and Na^+^/(Na^+^ + Ca^2+^). The second region, marked by moderate TDS levels, suggests the influence of rock weathering. The third region, located in the upper section, indicates evaporation and crystallization processes, characterized by extremely high salinity levels^28,34^. The plot reveals that water samples collected from all springs primarily fall within the rock weathering zone due to water rock interaction. However, all springs fell in similar zone regarding salinity but there is a horizontal trend increase from left to right in the Cl^−^/ (Cl^−^ + HCO_3_^−^) which could be influenced by the anthropogenic effect and reverse ion exchange process in the in Dobro Woda, Święto Woda, and ZimnySztok regions.

**Fig. 4:**
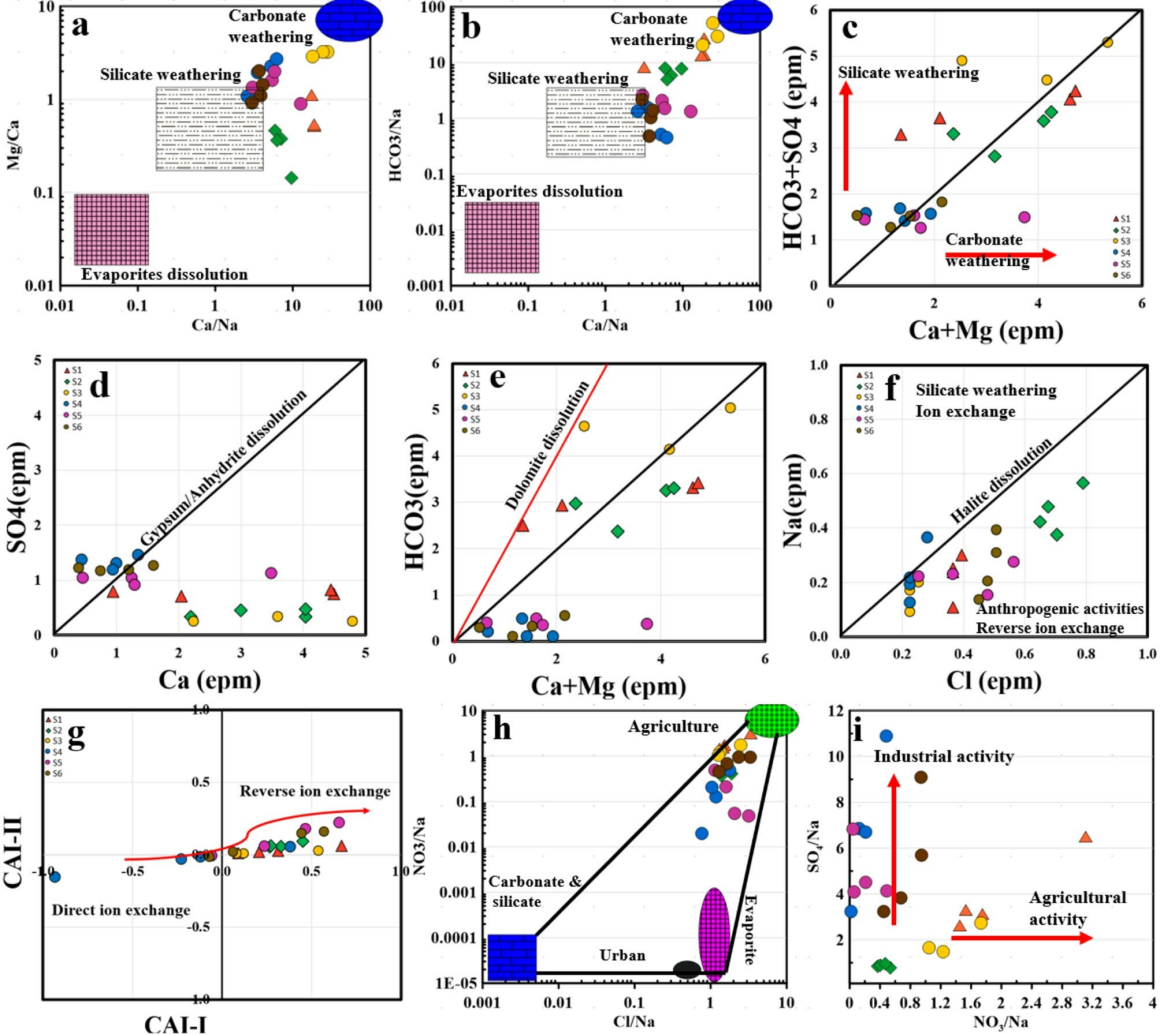
Ionic relationships: (**a**, **b**) Ca^2+^/Na^+^ against both Mg^2+^/Na^+^ and HCO_3_^−^/Na^+^, (**c**) Ca^2+^+ Mg^2+^ against SO_4_^2−^+HCO_3_^−^, (**d**) Ca^2++^ against SO_4_^2−^, (**e**) Ca^2+^+ Mg^2+^ against HCO_3_^−^, (**f**) Na^+^ vs. Cl^−^, (**g**) CAI-1 vs. CAI-2, (**h**, **i**) NO_3_^−^/Na^+^ against both SO_4_^2−^/Na^+^ and Cl^−^/Na^+^.

Figure 4a and b illustrate three potential mechanisms influencing water chemistry evolution based on the ionic ratios of Ca^2+^/Na^+^ vs. Mg^2+^/Na^+^ and Ca^2+^/Na^+^ vs. HCO_2__−_/Na^+^: evaporation-dissolution, silicate weathering, and carbonate weathering^92^.

Water samples from the Dobro Woda, Święto Woda, and Zimny Sztok springs revealed that silicate weathering is a significant factor control the water chemistry followed by carbonate weathering. while samples from Zygmunt, Leśniów, Halszka springs fell between silicate weathering and carbonate weathering. The geological formations situated within the vicinity of Dobro Woda, Święto Woda, and the Zimny Sztok springs constitute a structural entity recognized as the Upper Silesian block. These are Precambrian crystalline rocks, predominantly consisting of mica schists and paragneisses. Overlying them are Cambrian formations comprising siltstones and diabases, followed by Devonian deposits composed of sandstones transitioning into limestones, dolomites interspersed with claystone, mudstones, and marls. This sequence is succeeded by Lower Carboniferous strata characterized by sandstones, mudstones, shales, and conglomerates, with the upper Carboniferous segment containing carbonaceous sediments known for their productivity. The springs situated in Leśniów, namely the Zygmunt spring and the Halszka spring, are positioned within the Silesian-Cracow monocline. The sediments of the Lower and Middle Triassic epochs consist of sandstones, mudstones containing gypsum, and a diverse assemblage of carbonate rocks. The Lower Jurassic formations comprise gravels, sands, clays, mudstones, and sandstones. The Upper Jurassic formations consist of limestone layers exceeding 500 m in thickness. Quaternary sediments are predominantly found in valleys and depressions. These deposits mainly consist of sand and gravel derived from fluvial-glacial accumulations, sandy non-glacial deposits, and alluvial fan deposits. The geological composition could facilitate the silicate weathering to play significant role in evolution of water chemistry as well as dissolution, ion exchange, alongside carbonate weathering.

**Fig. 5:**
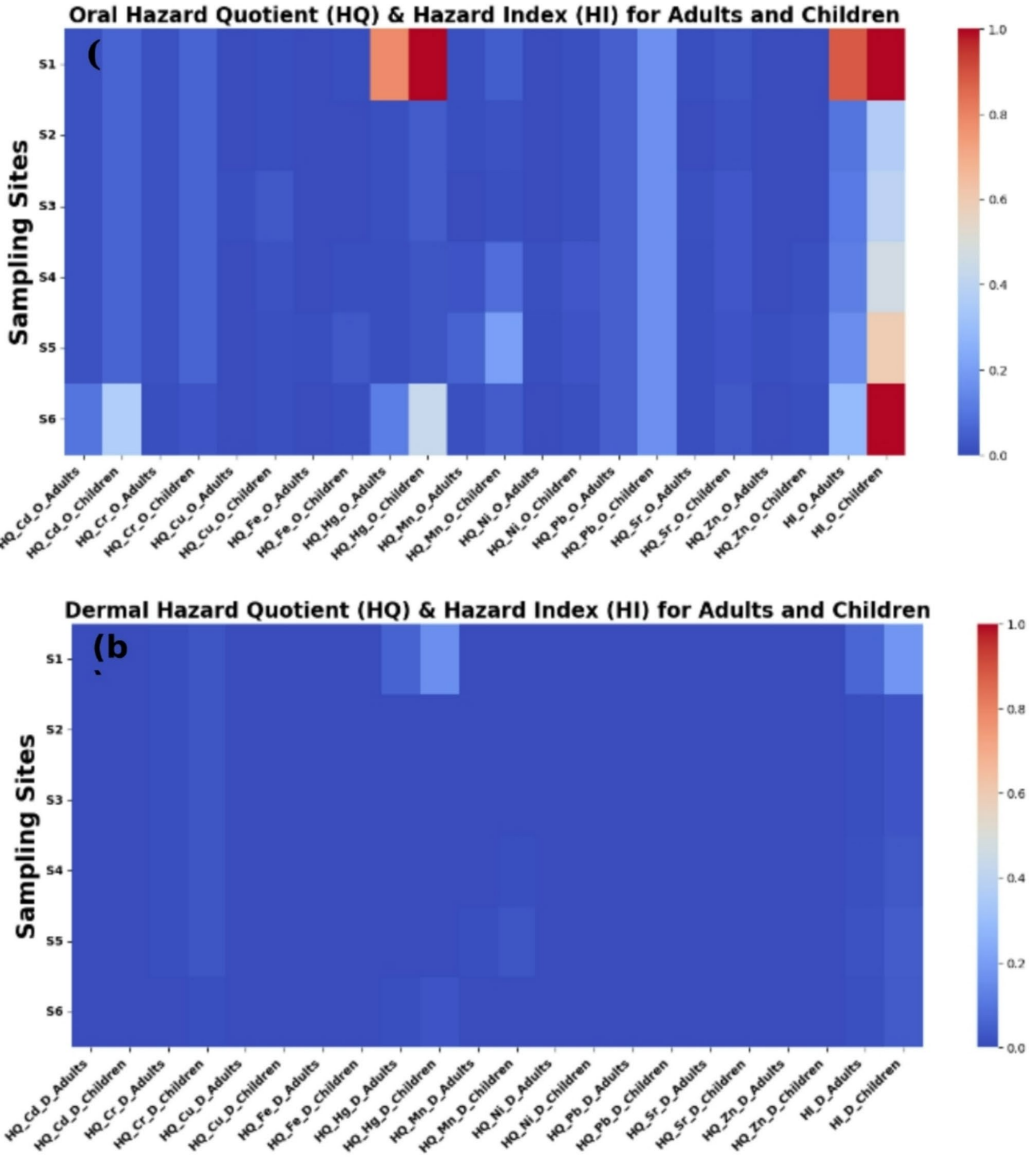
The Hazard Quotient and Hazard Index of the examined heavy metals: (**a**) oral and (**b**) dermal.

Plotting the springs samples above and below the 1:1 line for the relationship between Ca^2+^ + Mg^2+^ and HCO_2_^−^ + SO_4_^2−^ (Fig. 4c), confirm the significant role of both silicate and carbonate weathering in shaping the water chemistry of all springs. The crossing of the 1:1 line is attributed to the dissolution of gypsum, calcite, and dolomite. The week correlation between Ca and SO_4_ (Fig. 4d) demonstrated the anhydrite and gypsum as evaporite deposits are not the source of Ca and SO_4_ in the water samples which indicate that carbonate, silicate weathering as well as anthropogenic activities represent strong mechanism in evolution of water chemistry. The ratio of Ca^2+^ + Mg^2+^ to HCO_2_^−^ (Fig. 4e) can help identify the sources of calcium and magnesium in the samples (Fig. 5e). A ratio close to 0.5 suggests that Ca^2+^ and Mg^2+^ originate from the weathering of minerals^11,28,41,93^. A ratio below 0.5 may indicate ion exchange or bicarbonate enrichment as the cause of calcium and magnesium depletion. Most water samples exhibited a Ca^2+^ + Mg^2+^/HCO_2_^−^ ratio significantly higher than 0.5, with only a few samples close to this value. This implies that processes beyond carbonate dissolution, such as silicate weathering and reverse ion exchange, contribute to the elevated Ca^2+^ and Mg^2+^ levels. The moderately alkaline conditions and the lack of HCO_2_^−^ depletion suggest that silicate weathering and/or reverse ion exchange are the primary drivers of the high Ca^2+^ and Mg^2+^ concentrations. The plotting of the majority of samples below 1:1 line within the relationship between Na^+^ and Cl^−^ (Fig. 4f) may indicate either an enrichment of chloride from additional sources or a reduction in sodium levels due to Na^+^ removal from groundwater. Elevated chloride levels could stem from human activities like agricultural runoff, waste disposal, or atmospheric chloride deposition. To determine the type of ion exchange (direct or reverse), two Chloro-Alkaline Indices (CAI-I and CAI-II) were applied (Fig. 4g) a. Positive values of these indices indicate reverse ion exchange, while negative values suggest direct ion exchange according to the following two reactions (Eqs. 32, 33);

32 $$1/2{\text{Ca}}^{{2 + }} - {\text{X}} + {\text{Na}}^{ + } \to 1/2{\text{Ca}}^{{2 + }} + {\text{Na}}^{ + } - {\text{X}}\left( {{\text{reverseion}}\;{\text{ exchange}}} \right)$$

33 $${\text{Na}}^{ + } - {\text{X }} + {\text{ }}1/2{\text{Ca}}^{{2 + }} {\text{ }} \to {\text{ Na}}^{ + } {\text{ }} + {\text{ }}1/2{\text{Ca}}^{{2 + }} - {\text{X }}\left( {{\text{direction}}\;{\text{ exchange}}} \right)$$

The results demonstrated that the majority of springs samples exhibited reverse ion exchange, where Ca^2+^ and Mg^2+^ in the rock were replaced by Na^+^ and K^+^ in the water. The remaining few samples located in Dobro Woda showed negative CAI-I and CAI-II values, indicating direct ion exchange, where Ca^2+^ and Mg^2+^ in the water were replaced by Na^+^ and K^+^ in the rock.

Human activities have had a profound effect on water quality in the study area, largely due to the release of domestic wastewater and agricultural practices, which have led to increased levels of SO_4_^2−^, Cl^−^, and NO_2_^−^. The higher concentrations of SO_4_^2−^ and Cl^−^ in water samples are mainly attributed to the dissolution industrial and agricultural runoff, while NO_2_^−^ primarily stems from domestic sewage and farming activities. The ratios of Cl^−^/Na^+^ and NO_2_^−^/Na^+^ were employed to assess the impact of both human and natural activities on water quality and determine the source or origin of NO_3_^−^. As illustrated in Fig. 4h and i, all springs samples from six investigated regions, were influenced by agricultural practices as an input source of NO_3_^−^ in water. On the other hand, the relationship between SO_4_/Na vs. NO_3_/Na showed two anthropogenic sources of SO_4_ and NO_3_ in the springs.

It was found that the agricultural activities play important role in the Zygmunt, Leśniów, Halszka regions while the industrial activities were the significant factor of contaminations in the Dobro Woda, Święto Woda, and Zimny Sztok. This nitrate likely entered the groundwater through a series of steps: nitrogen gas (N_2_) infiltrated the aquifer via ancient precipitation, nitrogen-fixing bacteria converted it to ammonia (NH_2_), and nitrifying bacteria then transformed it into nitrate (NO_2_^−^)^94^. Samples from springs fell into an agricultural zone, where rising Cl^−^/Na levels and NO_2_^−^/Na ratios suggest that NO_2_^−^ in these samples came from a combination of soil nitrogen and agricultural sources. In contrast, springs samples from Dobro Woda, Święto Woda, and ZimnySztok exhibited very low NO_2_^−^/Na^+^ ratios and extremely high SO_4_/Na ratio, pointing to sewage as the primary source of NO_2_^−^ and SO_4_ leading to significant contamination.

### Geochemical modeling

PHREEQC modeling provides quantitative assessment of mineral saturation states through saturation indices (SI), which is essential for understanding the thermodynamic controls on water chemistry that cannot be determined through conventional hydrochemical analysis alone. This approach is particularly valuable in our study area where complex water-rock interactions occur across diverse geological formations. The calculation of saturation indices for minerals offers crucial insights into potential mineral precipitation or dissolution processes that may affect soil permeability and groundwater recharge^28,30^ in the study area. This predictive capability is essential for understanding long-term hydrogeological evolution in southern Poland’s spring systems.

**Fig. 6:**
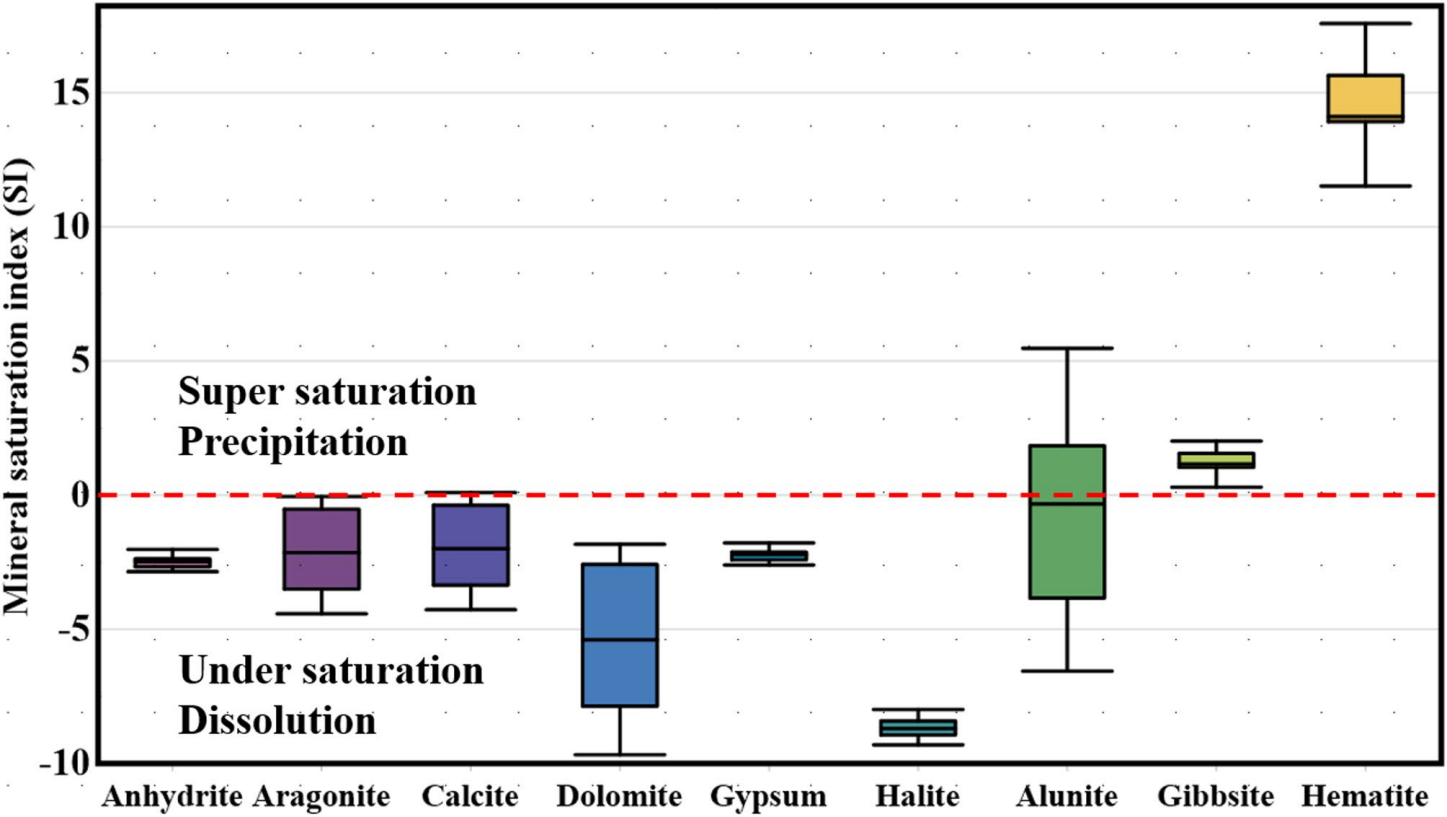
The extracted mineral phases from 6 springs and their response to dissolution or precipitation.

In this research, the saturation levels of minerals and salts in water were examined to determine which types of minerals might form deposits in the soil, potentially lowering soil permeability, and affecting the rate at which water seeps into the groundwater. The origins of chemical components were also explored using a model based on PHREEQC. Input data included measurements of physical and chemical properties, as well as concentrations of heavy metals. Saturation indices (SI) were computed for a range of minerals, such as calcite, Aragonite, gypsum, anhydrite, dolomite, Strontianite, Alunite, gibbsite, hematite, and Rhodochrosite, and displayed in a box plot (Fig. 6).

All water samples were below saturation (SI < 0) for anhydrite, halite, and gypsum, Calcite, Dolomite, and aragonite minerals, indicating that these minerals could continue to dissolve, further increasing water salinity. On the other hand, minerals like Alunite, hematite, Gibbsite were above saturation (SI > 0) in all samples (Fig. 6 and Table 4).

**Table 4:** The mineral phases extracted from PHREEQC model with saturation index values.

Phases/value	Min	Max	Mean
Anhydrite	−2.85	−2.02	−2.47
Aragonite	−4.42	−0.05	−2.08
Calcite	−4.27	0.1	−1.93
Dolomite	−9.08	−1.25	−4.68
Gypsum	−2.6	−1.78	−2.23
Halite	−9.31	−7.99	−8.66
Strontianite	−6.1	−2.2	−3.96
Alunite	−6.56	5.48	−0.78
Gibbsite	−0.77	2.6	1.26
Rhodochrosite	−5.17	−1.57	−3.29
Hematite	10.69	17.58	14.39

Gibbsite forms from the weathering of aluminosilicate minerals like feldspar or mica, releasing aluminum ions that react with hydroxyl ions in water. Hematite likely comes from the oxidation of iron-bearing minerals. The high saturation of water with these minerals can negatively affect soil permeability, fertility, water infiltration rates, and crop yields and further treatment is required before drinking or irrigation. Minerals hematite is often linked to hydrothermal systems and may indicate the presence of geothermal water at greater depths. Further study is needed, especially since the study area is in a tectonically active area. The SI index is a useful tool for predicting the geological makeup of the aquifer system, identifying the sources of primary and trace ions in surface and groundwater, and understanding mineralization processes. Beside the silicate weathering, Calcite, dolomite, aragonite, anhydrite, and gypsum, are likely contributed sources of Ca^2+^ in the water samples. Magnesium (Mg^2+^) may come from dolomite, and silicate minerals. Sodium (Na^+^) levels are likely influenced by halite and albite, while potassium (K^+^) may come from illite, K-feldspar, and K-mica. Hematite and chlorite are probable sources of iron (Fe), and rhodochrosite may contribute to manganese (Mn) levels. These findings highlight the complex interactions of geological and geochemical processes that shape water quality in the study area.

### Water quality index (CWQI)

**Fig. 7:**
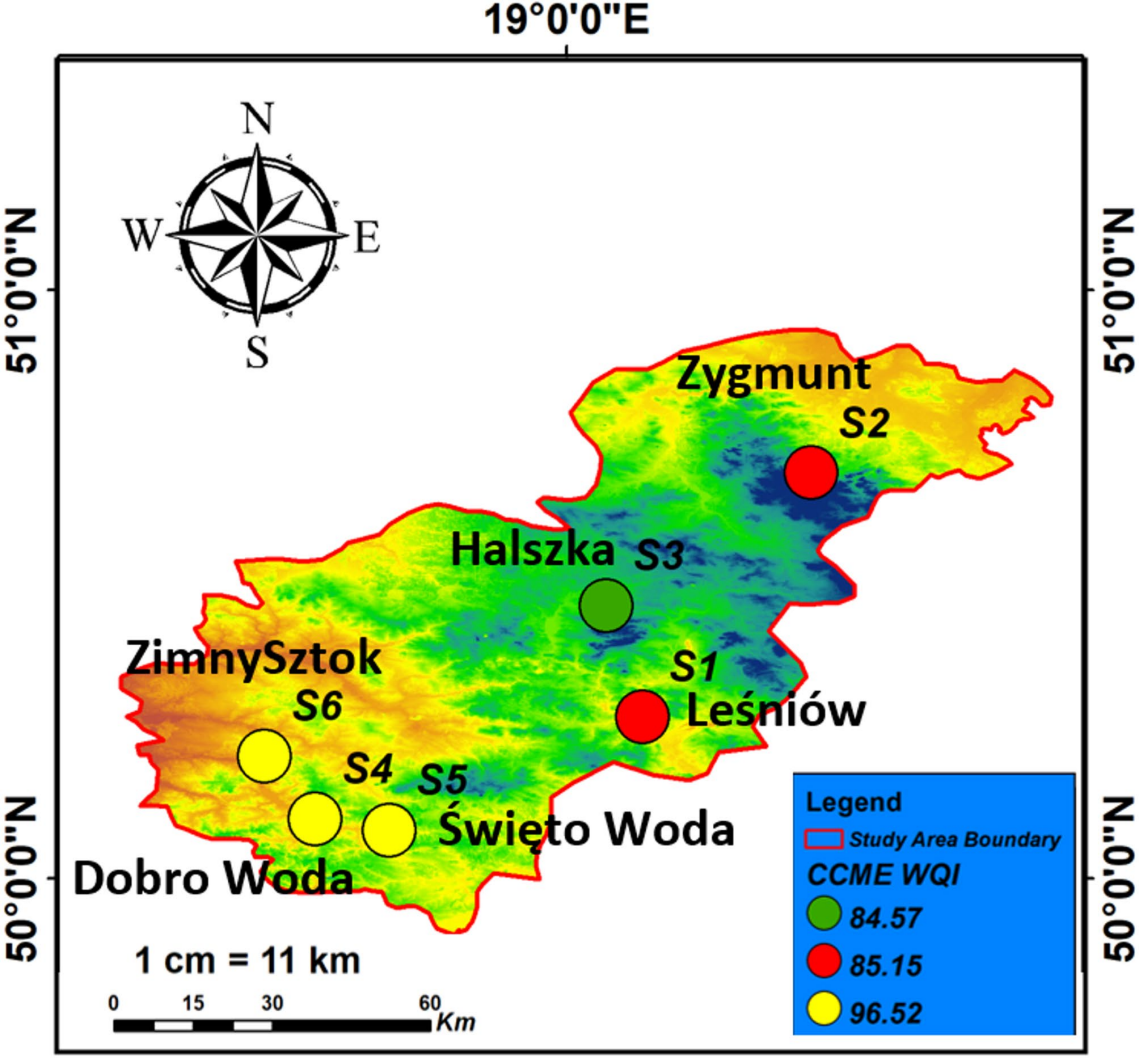
Water quality for drinking CCME WQI interpolated in the study area.

The CWQI methodology integrates multiple water quality parameters into a single numerical value, providing a holistic assessment that individual parameter analysis cannot achieve. This integration is essential for our study area where multiple potential contaminants from various sources may affect water quality simultaneously. The calculated CWQI values for the samples in this study all fall within the “Good” classification, indicating that the water quality at these locations is generally suitable for most uses, including drinking, aquatic life, and recreational purposes. The CWQI values range from 84.57 to 96.52, all of which are above the threshold for “Fair” quality, which starts at a CWQI of 60. This suggests that the water in these regions is well-managed, with minimal pollution or contamination (Fig. 7). Specifically, samples S1, S2, and S3, with CWQI values of 85.15, 85.15, and 84.57, respectively, show that the water quality at these sites is consistently good, with little to no indication of significant pollution or environmental stress. These values reflect a stable balance of water quality parameters, such as dissolved oxygen, pH, temperature, and concentrations of key contaminants, which are all within acceptable limits for supporting healthy ecosystems and safe water consumption.

The higher CWQI values of 96.52 for samples S4, S5, and S6 further confirm that the water quality at these locations is particularly high, falling into the upper range of the “Good” classification. These sites may be less impacted by anthropogenic activities and could benefit from natural environmental conditions that contribute to the preservation of clean water. These values suggest that these regions are less prone to contamination and have more favorable conditions for aquatic life and public health. Overall, the consistent “Good” classification across all sampling sites indicates that the water in the study area is of high quality, with no significant risks identified that would warrant immediate concern. However, continued monitoring is essential to maintain this status, as water quality can fluctuate due to seasonal changes, local pollution sources, or other environmental stressors. Long-term sustainability of water resources in these regions can be supported through regular assessments and adaptive management strategies to ensure water quality remains within acceptable limits for both human and ecological health.

By employing both hydrochemical facies analysis and the CWQI approach, our study provides a comprehensive assessment that integrates geochemical process understanding with practical water quality evaluation, offering valuable insights for both scientific understanding and water resource management in southern Poland.

### Heavy metal pollution index (HPI) and heavy metal index (HMI)

**Fig. 8:**
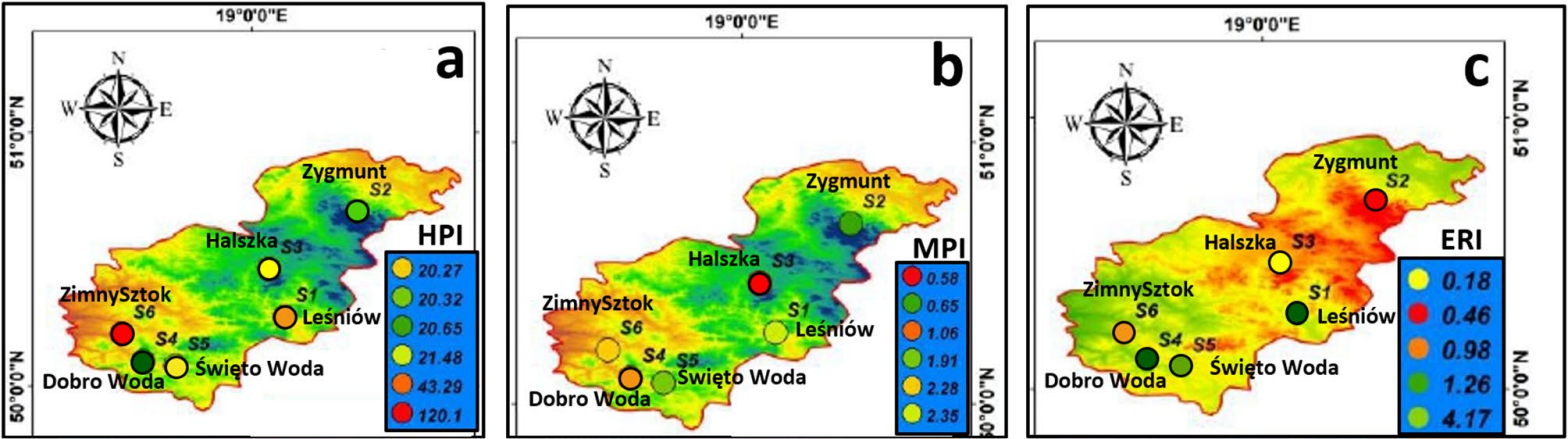
Contamination indices: (**a**) HPI; (**b**) HMI; and (**c**) ERI in southern Poland springs.

The HPI values of the study area ranging from 20.27 to 120.10. Among these, S1 has the highest HPI value of 43.29, indicating a relatively moderate level of heavy metal contamination. The values for S2 (20.32), S3 (20.27), S4 (20.65), and S5 (21.48) are quite similar, all falling within a narrow range of approximately 20 to 21, suggesting relatively low levels of contamination in these samples. However, S6 stands out significantly with an HPI value of 120.10, which is much higher than the rest and indicates a far greater level of heavy metal pollution. This sharp contrast between S6 and the other samples suggests that there may be an unusual contamination event or an anomaly in the sampling at S6 (Fig. 8a). Given the relatively lower HPI values of the other samples, it is essential to investigate the source and nature of the pollution at S6, as it may represent a localized area of significant contamination. This variation in HPI values highlights the need for further examination to identify specific pollution sources and assess potential risks to environmental and human health. However, the MI values ranging from 0.58 to 2.35. S1has the highest MI value of 2.35 indicating a relatively high level of metal pollution in this sample. The MI values for S2 (0.65), S3 (0.58), and S4 (1.06) are notably lower, suggesting that these samples are relatively less contaminated with metals. S5, with an MI value of 1.91, shows a significant increase in metal contamination compared to S2, S3, and S4, but still remains lower than S1. Finally, S6 has an MI value of 2.28, which is just slightly lower than S1, indicating another site with relatively high metal pollution (Fig. 8b). Overall, the MI values suggest varying degrees of metal pollution across the sampling locations, with S1and S6 standing out as the most heavily polluted sites. These variations highlight the need for targeted interventions at sites with higher MI values to reduce metal contamination and mitigate potential environmental and health risks. Further investigation into the sources of pollution at the more contaminated sites, such as S1and S6, is essential for developing effective management strategies.

### Ecological risk index (ERI)

The RI in this study shows values ranging from 0.18 to 4.17, suggesting that the majority of the sampled locations pose relatively low ecological risk (Fig. 8c). S1 and S4 have identical ERI values of 1.26, both falling within the low-risk category (ERI < 30), which indicates minimal environmental risk. Similarly, S2 (0.46), S3 (0.18), and S6 (0.98) also fall into the low-risk category, further emphasizing that the ecological risk in these locations is relatively insignificant. However, S5 stands out with a higher ERI value of 4.17, but it still remains within the low-risk range. This suggests that while there is an elevated risk compared to other samples, it does not reach a level that would indicate significant environmental concern. In the context of the risk indicator classification, all the samples in this dataset fall under the low-risk category (ERI < 30), with no sites classified as having moderate or higher risk. These results imply that the ecological impact of heavy metal contamination in the groundwater of the studied area is generally low. However, it remains important to continue monitoring the samples, particularly S5, as even lower levels of risk may accumulate over time or under certain conditions, potentially escalating into more significant environmental issues. Overall, these findings suggest that the groundwater quality in this region is not currently at a level that poses serious ecological threats, but continued vigilance is essential to ensure the sustainability of the environment.

### Human health risk assessment

#### Non-carcinogenic health risk

At all six sampling sites, both oral and dermal health risks associated with heavy metal exposure show significant differences between adults and children, with children consistently facing higher risks. For oral exposure (Fig. 5a), at Sampling Site S1, the HQ values for the studied heavy metals reveal significant differences between adults and children. For Cd and Cr, both adults and children show similar HQ values (1.51 E−02 for adults and 5.75 E−02 for children), indicating a low level of risk. For Cu and Fe, the HQ values are relatively low as well, with Cu showing 1.51 E−03 for adults and 5.75 E−03 for children, and Fe having values of 1.25 E−03 for adults and 4.77 E−03 for children. However, Hg stands out with much higher HQ values, showing 7.87 E−01 for adults and 3.01 E + 00 for children, indicating a significant health risk, especially for children. For Mn, adults exhibit a HQ of 1.07 E−02, while children have 4.08 E−02, highlighting a much higher risk for children. Similarly, Ni values are higher for children (1.15 E−02) compared to adults (3.01 E−03). Pb also shows a higher risk for children, with an HQ of 1.64 E−01, compared to 4.31 E−02 in adults. For Sr, the HQ values for children (1.82 E−02) are higher than those for adults (4.77 E−03), and Zn values also show a higher risk for children (3.45 E−03) compared to adults (9.04 E−04). Overall, the HI for adults at S1 is 8.83 E−01, while for children, it significantly increases to 3.37 E + 00, suggesting that children are more vulnerable to the health risks associated with heavy metal exposure in this area. At Sampling Site S2, the trend remains largely similar, with Cd and Cr showing consistent low-risk values for both groups. However, metals like Hg, Mn, and Pb again demonstrate higher HQ values for children. Specifically, Hg reaches 3.55 E−02 for children compared to 9.29 E−03 for adults, while Pb reaches 1.64 E−01 for children, indicating a higher level of exposure and risk for children. The HI for children at this site is 3.67 E−01, higher than the HI for adults (9.61 E−02), reinforcing the heightened risk for children. At Sampling Site S3, similar patterns are observed with Cd and Cr continuing to show low-risk values, while Hg, Mn, and Pb exhibit higher HQ values for children. Notably, Hg reaches 3.58 E−02 for children, and Pb reaches 1.64 E−01. The HI for children (3.93 E−01) remains higher than that of adults (1.03 E−01). In Sampling Site S4, the HQ values for Cd, Cr, and Cu are similar to those in the previous sites, with Pb again showing a higher risk for children. The HI for children (4.63 E−01) is substantially higher than for adults (1.21 E−01), indicating a notable increase in health risks at this site. At Sampling Site S5, the risk for children continues to be greater, especially for metals such as Mn (5.27 E−02 for children vs. 2.01 E−01 for adults) and Pb (1.64 E−01 for children vs. 6.04 E−03 for adults). The HI for children (5.94 E−01) remains significantly higher than for adults (1.56 E−01). Finally, at Sampling Site S6, the HQ values for Mn and Pb continue to show significantly higher risks for children compared to adults, with Pb reaching 1.08 E + 00 for children, which is a considerable concern. The HI for children at this site is 1.08 E + 00, much higher than the HI for adults (2.82E −01), again indicating increased vulnerability to heavy metal toxicity in children. Overall, these results indicate that across all six sampling sites, children consistently face higher health risks from heavy metals than adults, particularly from metals such as Hg, Mn, and Pb, emphasizing the need for targeted measures to reduce children’s exposure to these potentially toxic substances.

For dermal exposure (Fig. 5b), a similar trend emerges. At Sampling Site S1, the dermal HQ values for the studied heavy metals exhibit notable variations between adults and children. For Cd, Cr, and Cu, both adults and children show similar low HQ values, with Cd at 3.58 E−05 for adults and 1.05 E−04 for children, and Cr at 5.72 E−03 for adults and 1.69 E−02 for children. Cu exhibits relatively low HQ values for both groups as well (2.38 E−05 for adults, 7.03 E−05 for children). However, for Hg, the dermal exposure risk is higher, with Hg showing 5.34 E−02 for adults and 1.57 E−01 for children, indicating a greater risk for children. Similarly, Mn shows a higher risk for children (3.74 E−03 for adults, 1.27 E−03 for children). Ni and Pb show similar trends, with children exhibiting higher dermal exposure risks compared to adults (Ni at 7.15 E−05 for adults and 2.11 E−04 for children, Pb at 6.81 E−05 for adults and 2.01 E−04 for children). Sr and Zn show relatively low dermal exposure risks, but children still have higher values for both metals (Sr at 1.13 E−04 for adults and 3.34 E−04 for children, Zn at 1.29 E−05 for adults and 3.80 E−05 for children).

The Hazard Index (HI) for adults at S1 is 6.07 E−02, while for children, it is 1.79 E−01, showing a significant increase in risk for children due to dermal exposure to these heavy metals. At Sampling Site S2, the dermal HQ values for Cd, Cr, and Cu remain consistent with S1, showing similar low-risk values. However, Hg again shows a higher risk for children (1.86 E−03 for children vs. 6.30 E−04 for adults). For Mn, the HQ values are slightly higher for both groups compared to S1, with children exhibiting greater exposure risk (4.66 E−04 for adults, 1.37 E−03 for children). Similarly, Ni and Pb values remain higher for children (Ni at 7.15 E−05 for adults and 2.11 E−04 for children, Pb at 6.81 E−05 for adults and 2.01 E−04 for children). The HI for adults at S2 is 7.13 E−03, while for children, it is 2.10 E−02, indicating an increased risk for children.

At Sampling Site S3, the trend continues, with Cd and Cr showing low dermal exposure risk for both groups. However, for Cu, Mn, Ni, and Pb, the dermal HQ values are elevated for children. Specifically, Pb reaches 2.01 E−04 for children compared to 6.81 E−05 for adults. Hg also shows a higher risk for children (1.88 E−03 for adults, 6.36 E−04 for children). The HI for adults is 7.09 E−03, and for children, it is 2.09 E−02, continuing the pattern of greater risk for children. At Sampling Site S4, the dermal exposure risk for children continues to be elevated, especially for metals like Hg (1.00 E−03 for children vs. 3.41 E−04 for adults) and Mn (7.14 E−03 for children vs. 2.42 E−03 for adults). The HI for adults is 9.08 E−03, while for children, it increases to 2.68 E−02, further indicating the heightened risk for children. At Sampling Site S5, similar trends are observed, with Hg, Mn, and Pb posing greater dermal exposure risks for children. The HI for adults is 1.30 E−02, and for children, it is 3.82 E−02, confirming that children continue to face higher dermal health risks. Finally, at Sampling Site S6, the highest dermal exposure risks for children are observed for Hg (2.29 E−02 for children vs. 7.75 E−03 for adults) and Mn (3.52 E−03 for adults vs. 1.19 E−03 for children). The HI for adults is 1.17 E−02, while for children, it increases to 3.45 E−02, further highlighting the vulnerability of children to dermal exposure to these metals. In conclusion, at all six sampling sites, children consistently exhibit higher dermal health risks from heavy metals compared to adults, particularly from Hg, Mn, and Pb. This underscores the need for heightened protective measures to reduce children’s exposure to these hazardous substances.

#### Monte Carlo simulation approach

The Monte Carlo simulation approach was employed to estimate the HQ values for oral and dermal exposure to the heavy metals: Fe, Cu, Mn, Cr, Ni, Cd, Hg, Sr, Zn, and Pb. Additionally, the simulation was used to assess the carcinogenic risk (CR) associated with oral and dermal exposure to Cr and Pb for both adults and children. This method allows for the incorporation of variability and uncertainty in exposure data, providing a more comprehensive risk assessment for the target populations.

#### Non-carcinogenic risk assessment

**Fig. 9:**
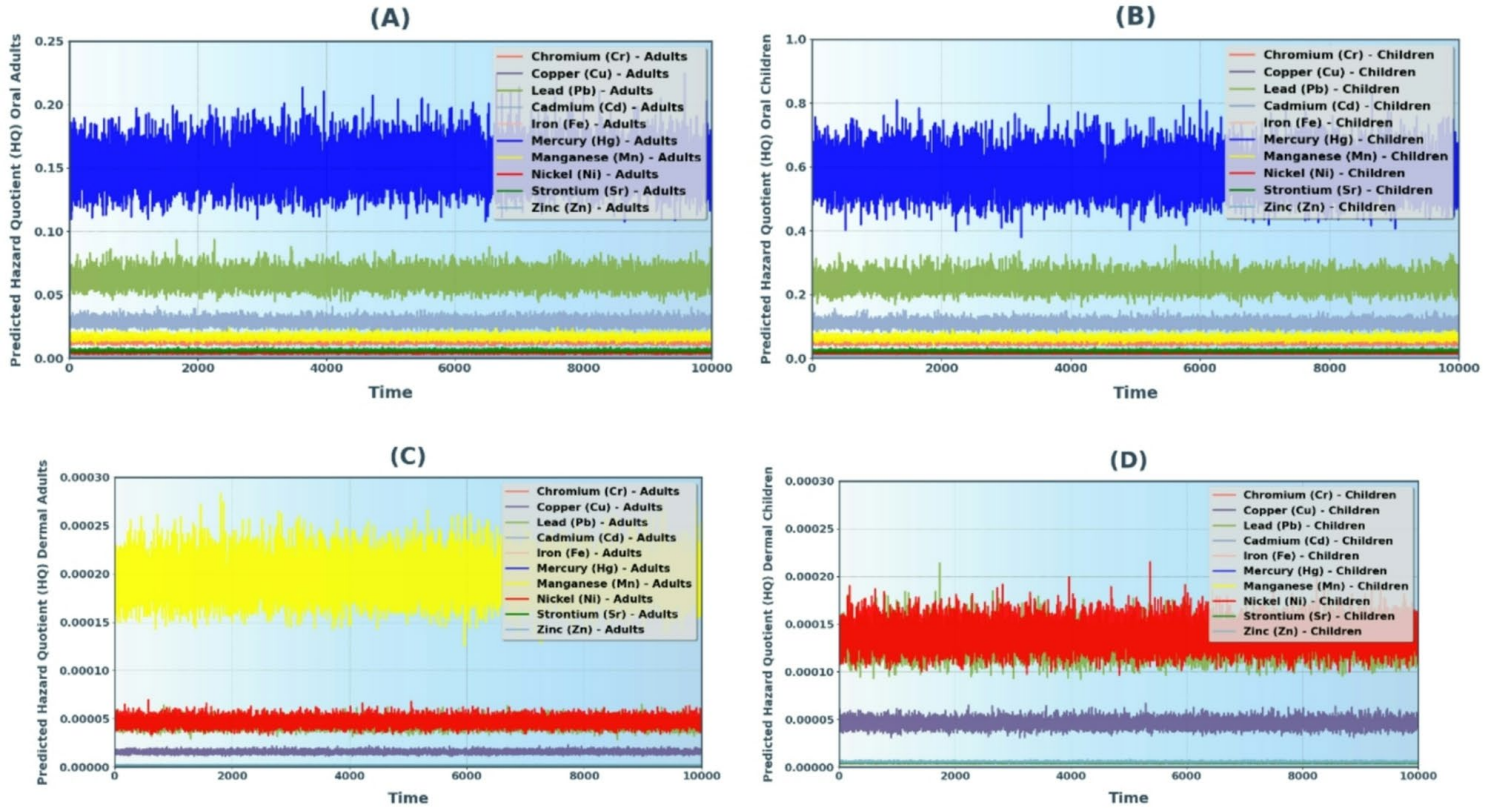
The oral and dermal predicted hazard quotient (HQ) for adults and children.

The Monte Carlo simulation results revealed that the predicted oral Hazard Quotient (HQ) values for adults across all assessed heavy metals (Fe, Cu, Mn, Cr, Ni, Cd, Hg, Sr, Zn, and Pb) remained consistently below the standard safety threshold (HQ < 1) (Fig. 9), indicating a manageable risk level for the adult population. This suggests that the estimated exposure levels are unlikely to pose a significant health risk through oral exposure for either adults or children. Similarly, both oral and dermal contact pathways were found to present minimal risk. However, it is important to acknowledge that risk assessments are often based on conservative assumptions and the inherent uncertainties in the available data. Consequently, ongoing monitoring of exposure levels is crucial, and risk assessments should be periodically updated to incorporate new information and address emerging risks^60–62,64^.

#### Carcinogenic health risk assessment

In this study, the MCS was employed to model carcinogenic health risks, building on deterministic risk assessment methods for both oral and dermal exposure pathways in adults and children. The analysis utilized the 5th and 95th percentiles of exposure data to represent the best-case and worst-case risk scenarios, respectively.

#### Chromium (Cr)

**Fig. 10:**
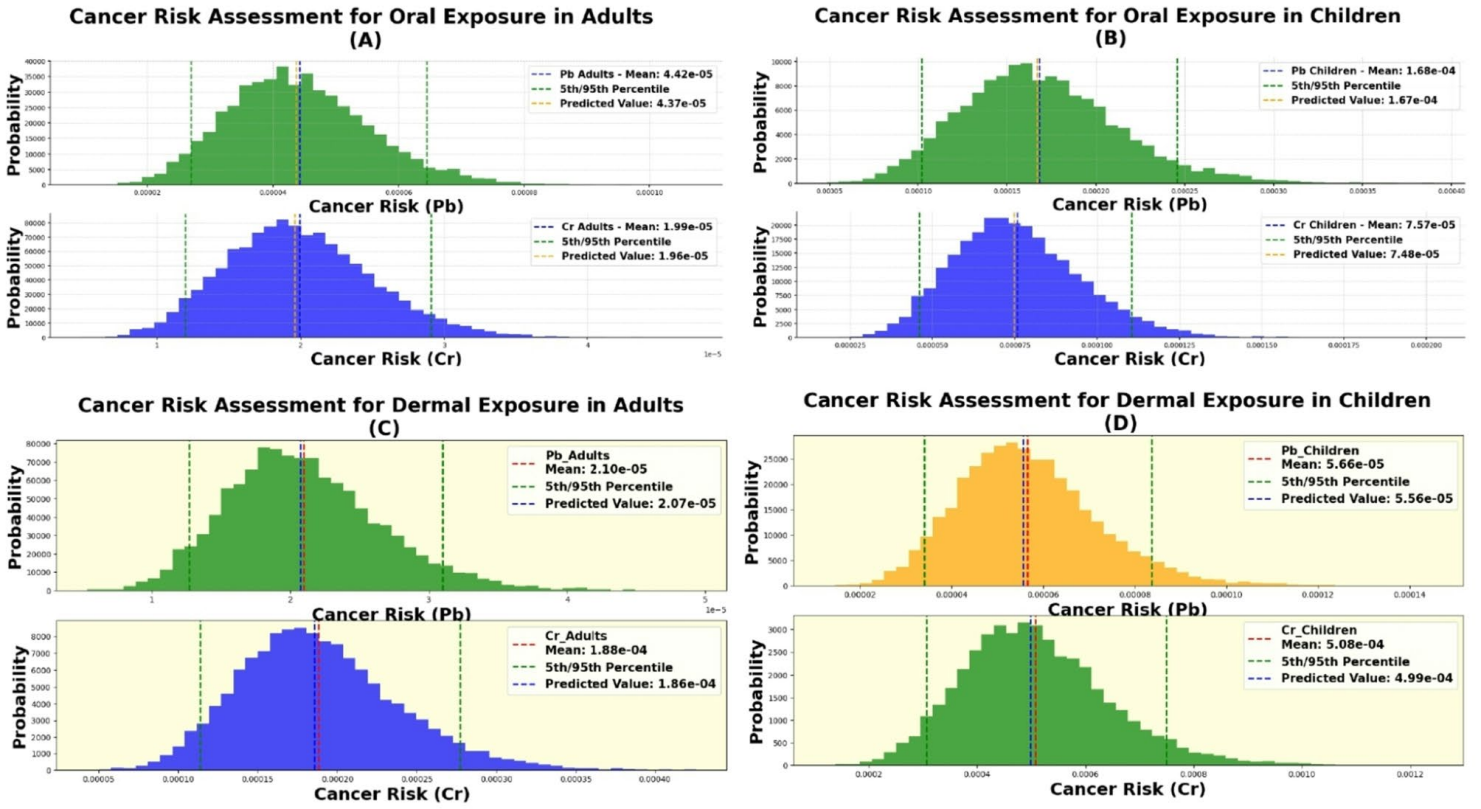
Carcinogenic risk: (**A**) oral exposure in adults; (**B**) oral exposure in children; (**C**) dermal exposure in adults; and (**D**) dermal exposure in children.

The carcinogenic risk from Cr exposure was assessed for both oral and dermal pathways in adults and children (Fig. 10). For oral exposure, the estimated cancer risk in adults ranged from a 5th percentile value of 1.21E−5 to a 95th percentile value of 2.87 × 10^−5^These values remain well below the acceptable risk range of 1.0 × E−6 to 1E−4, suggesting minimal carcinogenic risk for adults. For children, the oral cancer risk ranged from 4.68 × E−5 to 1.10 E−4, with the upper bound slightly exceeding the threshold, indicating higher potential risk in worst-case exposure scenarios. For dermal exposure, the cancer risk estimates in adults ranged from 1.1E−4 to 2.7E−4, exceeding the lower threshold and indicating significant risk. Children exhibited even higher dermal cancer risk values, ranging from 3.0 E−4 to 7.40 E−4, highlighting increased vulnerability in younger populations. These findings underscore the need for targeted mitigation efforts, particularly for children^60,62,64,95^.

#### Lead (Pb)

Lead exposure also presented carcinogenic risks, assessed for both exposure pathways (Fig. 10). For oral intake, the estimated cancer risk in adults ranged from 2.70E−5 to 6.45E−5, which is within the acceptable risk range. In children, values ranged from 1.0 E−4 to 2.40 E−4, indicating exceedance of the lower risk threshold in some cases. For dermal exposure, adult cancer risk estimates ranged from 1.25 × E−5 to 3.07 E−5, remaining below the concern threshold. However, children showed slightly higher dermal risk values ranging from 3.39 E−5 to 8.33 E−5. These results confirm that children face greater carcinogenic risks from Pb exposure, particularly through ingestion. Therefore, proactive measures to reduce environmental lead concentrations are critical to safeguard child health^60,62,64^.

#### Cumulative carcinogenic risk assessment

To better reflect the overall health burden from multiple contaminants, we calculated the cumulative carcinogenic risk from combined exposure to chromium (Cr) and lead (Pb) through the dermal pathway in adults. Using results from the Monte Carlo simulation (*n* = 10,000), the mean cumulative cancer risk was found to be 2.10 E−4, with the 5th and 95th percentiles ranging from 1.27 E−4 to 3.07 E−4. These values surpass the acceptable risk threshold set by the United States Environmental Protection Agency (USEPA), which considers cancer risks in the range of 1.0 E−6 to 1.0 E−4 as tolerable. This indicates that individuals exposed to both contaminants simultaneously face a significantly elevated carcinogenic risk. The findings emphasize the need for integrated mitigation strategies that account for cumulative risks rather than evaluating contaminants in isolation.

### Corrosion and scaling potentials (CSPs) indices

#### Langelier saturation index (LSI)

**Fig. 11:**
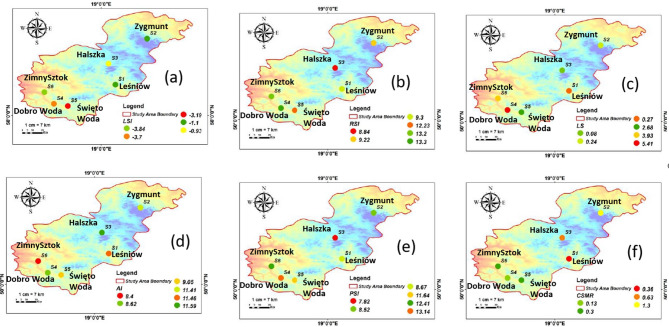
CSPs indices: (**a**) LSI; (**b**) PSI; (**c**) AI; (**d**) LS; (**e**) CSMR; and (**f**) RI.

The LSI represents the degree of calcium carbonate (CaCO₃) saturation in water. A negative value indicates that the water is undersaturated with respect to CaCO₃ and may promote corrosive behavior^96^. In this study, LSI values range from − 3.84 to −0.93, with all samples showing negative values, suggesting a tendency toward corrosive conditions (Fig. 11a). For instance, samples S4 (LSI = −3.70) and S6 (LSI = −3.84) exhibit the lowest values, indicating a high propensity for corrosive behavior due to the significant undersaturation with calcium carbonate. On the other hand, samples like S1 and S2 (both with LSI = −1.10) have a moderate negative LSI, also pointing toward corrosion but to a lesser degree. The negative LSI values across all samples reinforce the general tendency toward corrosion in the studied water sources, with no samples showing signs of scale formation, as all values are below zero.

#### Ryznar stability index (RSI)

The RSI assesses the potential for calcium carbonate scale formation or corrosion. Higher RSI values indicate a stronger tendency towards scaling or corrosion^96^. The RSI values in this study range from 8.84 to 13.30 (Fig. 11b). Most samples, including S4 (RSI = 13.30) and S6 (RSI = 13.20), show significantly high values, suggesting a pronounced risk of corrosion. These elevated RSI values, well above the threshold of 6.8, reflect a clear tendency for the water to be corrosive. Sample S3 (RSI = 8.84) falls at the lower end of the corrosive range but still indicates a moderate to high tendency for corrosion. The high RSI values suggest that the water in this dataset is generally unstable and highly prone to corrosion rather than scale formation.

#### Aggressive index (AI)

The AI is another measure of water’s corrosive potential, especially with respect to asbestos pipes. Values below 10 indicate highly aggressive water, while those above 12 signal non-aggressive, stable waters. The AI values in this study range from 8.40 to 11.59 (Fig. 11b). Samples like S3 (AI = 11.59) and S2 (AI = 11.41) show moderate values, indicating a low to moderate risk of corrosion. However, samples such as S4 (AI = 8.62) and S6 (AI = 8.40) have values below 10, signifying a highly aggressive water chemistry. These lower values indicate a higher corrosive potential, suggesting that measures should be taken to control the corrosion risk, particularly in regions with AI values nearing or below 10 ^97,98^.

#### Puckorius scaling index (PSI)

The PSI helps assess the potential of water to either form scale or promote corrosion^97^. Generally, PSI values above 6 indicate a corrosive tendency, while values below 6 suggest scaling potential. In this study, PSI values range from 7.82 to 13.14, supporting the interpretation of a predominantly corrosive water chemistry (Fig. 11c). For instance, sample S3 (PSI = 7.82) and sample S6 (PSI = 12.41) both fall within the corrosive range, consistent with the findings from LSI, RSI, and AI. These results collectively confirm that the studied waters exhibit a clear corrosive nature, with minimal risk of scale formation.

#### Revelle index (RI)

The RI measures the influence of salinization processes on water chemistry. It reflects the water’s ability to resist changes in pH with additional CO_2_ and is used to assess the buffering capacity of water^99^. In this study, RI values range from 0.03 to 0.90 (Fig. 11d), showing varying degrees of buffering. For instance, S3 (RI = 0.03) and S1 (RI = 0.07) exhibit low RI values, indicating that the waters are less influenced by salinization and are more likely to be sensitive to changes in CO_2_ concentrations. In contrast, sample S6 (RI = 0.90) has a much higher value, reflecting a stronger buffering capacity against pH changes. The majority of the samples (S2, S4, and S5) show moderate RI values, indicating moderate salinization effects and an intermediate buffering capacity.

#### Chloride-Sulfate mass ratio (CSMR)

The CSMR is important for assessing the corrosive potential of water, particularly for metal pipes. Higher chloride levels relative to sulfate levels generally enhance corrosivity^98^. In this study, CSMR values range from 0.13 to 1.64. Samples such as S4 (CSMR = 0.13) and S5 (CSMR = 0.30) exhibit lower CSMR values (Fig. 11e), suggesting a lower potential for corrosion due to the dominance of sulfate over chloride. In contrast, samples like S2 (CSMR = 1.30) and S1 (CSMR = 0.36) show higher ratios, indicating a greater likelihood of corrosion. The higher chloride levels in these samples suggest that the water is more corrosive, particularly in metal infrastructure, and may require additional treatment or protection strategies.

#### Larson-Skold index (LS)

The LS is a metric used to assess the corrosivity of water, especially concerning its effect on ferrous materials^96,99^. The LS values in this study range from 0.08 to 5.41(Fig. 11f). Samples such as S1 (LS = 0.27) and S2 (LS = 0.24) show low values, suggesting minimal corrosivity concerning chloride and sulfate interactions with bicarbonates. However, other samples like S4 (LS = 5.41) exhibit significantly higher LS values, indicating a greater risk of corrosion, particularly to ferrous materials. The higher LS values suggest that localized corrosion, including pitting corrosion, could occur, and additional corrosion control measures should be considered. Overall, these findings indicate that the water sources exhibit varying levels of corrosivity, with some samples showing high corrosion risks (S4, S6) while others exhibit relatively low to moderate risks. The variability in the indices highlights the complex nature of water chemistry and the need for tailored treatment strategies depending on the specific water quality parameters of each region.

### Practical applications of findings and policy frameworks

The findings provide a foundation for evidence-based water management policies. The consistently “Good” CWQI values (84.57–96.52) across sampling sites suggest current water management practices are effective, but continuous monitoring systems should be institutionalized to maintain this quality. Site-specific policies are warranted based on contamination indices: ZimnySztok spring exceptionally high HPI (120.10) and Leśniów and ZimnySztok springs elevated MI values (2.35 and 2.28) require targeted remediation strategies and stricter discharge regulations for nearby industrial activities.

The health risk assessment reveals critical policy implications, particularly for protecting vulnerable populations. Children consistently demonstrated higher non-carcinogenic risk across all sites, with HI values up to 3.37 for oral exposure at Leśniów and concerning Pb levels at S6 (HQ = 1.08). This necessitates child-specific safety standards in water quality regulations and enhanced filtration systems in schools and residential areas near high-risk sites. The study recommends implementing: (1) differential water quality standards based on demographic vulnerability; (2) mandatory heavy metal monitoring programs focusing on Hg, Pb, and Mn; (3) zoning regulations restricting industrial activities near water sources with elevated contamination indices; and (4) public health interventions targeting children’s exposure in high-risk areas, particularly S1 and.

To mitigate corrosion, chemical stabilization should be implemented by adjusting the pH to 7.5–8.5 using lime or soda ash, and adding orthophosphate inhibitors such as zinc orthophosphate to encourage the formation of a protective calcium carbonate (CaCO₃) scale, aiming for a target LSI above − 0.5. For springs with high chloride-to-sulfate mass ratios (CSMR) and low stability (e.g., S2 and S4), ion exchange or nanofiltration should be used to reduce Cl^−^/SO_4_^2+^ ratios below 0.5. In areas with elevated corrosion risk (LS > 5, RSI > 10), ferrous pipelines should be replaced with corrosion-resistant materials such as HDPE-lined ductile iron. Continuous monitoring of LSI and RSI using real-time sensors, along with automated dosing systems, is recommended for dynamic and responsive corrosion control. These targeted strategies address the primary corrosion factors low LSI, high RSI, and CSMR while responding to the extreme corrosivity observed in springs like S4 and S6.

## Limitation of the study and future work

### Limitation

The study was conducted at six spring locations, which, while representative of the southern Poland region, may not capture the full hydrogeochemical diversity of all spring systems in the area. Seasonal sampling was limited to two distinct periods (winter and summer), and more frequent sampling throughout the year could provide better understanding of temporal variations. The study focused primarily on major ions and selected heavy metals, and future work could benefit from analysis of emerging contaminants and trace organic compounds. Arsenic (As) and Cadmium (Cd) are well-known priority contaminants in groundwater due to their toxicity and carcinogenic potential, as extensively documented in the literature. However, in the present study, we focused our risk assessment specifically on Chromium (Cr) and Lead (Pb), as these were the only heavy metals consistently detected at quantifiable levels across all sampled sites. Due to time and resource limitations, as well as the absence of reliable or complete data for Arsenic and Cadmium during the sampling campaign, these elements were not included in the current risk modeling and analysis and can be investigated in the future work.

### Future work

Expansion of the monitoring network to include additional spring locations across different geological settings. Implementation of continuous monitoring systems to capture short-term variations in water chemistry. Investigation of isotopic signatures to better understand recharge sources and water-rock interaction processes. Development of predictive models for seasonal water quality variations to support management decisions. Assessment of climate change impacts on spring water quality and flow characteristics.

## Conclusion

This study elucidates the seasonal hydrogeochemical dynamics and anthropogenic influences on groundwater quality across six springs in southern Poland. The key findings highlight the interplay of geochemical processes, human activities, and seasonal variability in shaping water quality. Seasonal fluctuations in major ions (Ca^2+^, Mg^2+^, HCO_2_^−^, SO_4_^2−^, Cl^−^) are primarily driven by mineral weathering especially carbonate and silicate dissolution and evaporative concentration. These variations exhibit distinct patterns between wet and dry seasons. Anthropogenic inputs, particularly of NO_2_^−^, Cl^−^, and SO_4_^2−^, are pronounced in springs such as Dobro Woda, Święto Woda, and Zimny Sztok. Here, the presence of mixed Ca–Mg–Cl/SO_4_ facies and elevated ionic ratios (NO_2_^−^/Na^+^ > 1) strongly suggest contamination from agricultural and industrial sources. Additionally, heavy metals such as Fe, Mn, and Pb show peak mobilization during the summer, likely linked to redox-driven release from sediments and increased surface runoff from anthropogenic sources.

In terms of resource protection, the springs dominated by Ca–Mg–HCO_2_ facies (Zygmunt, Leśniów) reflect a strong natural buffering capacity due to rock weathering. However, springs with mixed facies require targeted monitoring for Cl^−^ and SO_4_^2−^ pollution, especially where local agriculture or industry exerts significant pressure. From a public health perspective, the detection of chronic Pb exposure (hazard quotient (HQ) > 1 for children at Zimny Sztok spring) and elevated carcinogenic risk from Cr (risk > E−4 via dermal exposure) underscore the urgent need for water filtration systems in vulnerable areas and tighter regulation of metal discharges. Infrastructure concerns are also raised, as persistently negative Langelier Saturation Index (LSI values between − 3.84 and − 0.93) and high Ryznar Stability Index (RSI > 8.8) point to corrosive water chemistry, calling for the use of corrosion-resistant materials in local water distribution networks.

To improve contamination source tracking, the application of isotopic tracers such as δ^15^N–NO_2_^−^ and δ^24^S–SO_4_^2−^ is recommended for differentiating between agricultural and industrial pollution in mixed-facies springs. Seasonal monitoring should be intensified, particularly after the summer period, to effectively capture peaks in metal and ion concentrations due to evaporation. These insights bridge site-specific observations with broader global challenges in groundwater management, emphasizing the complex interactions between lithology, climate variability, and anthropogenic pressures. Future studies should aim to quantify contaminant fluxes across different phases of the hydrological cycle to support more effective mitigation strategies.

## Electronic supplementary material

Below is the link to the electronic supplementary material.

Supplementary Material 1

## Data Availability

The datasets utilized and/or analyzed during the current study are available in the manuscript and supplementary material submitted with the manuscript.
