# Supplementary material for: Seasonal hydrochemical characteristics of spring water in Southern Poland: integrating geochemical modeling, health risk analysis and mitigation strategies
Source: Sci Rep. 2025 Jul 15;15:25459. doi: 10.1038/s41598-025-10322-5 (PMC12259870; doi:10.1038/s41598-025-10322-5)
Supplement: Supplementary file 1 — Supplementary Material 1 [file 41598_2025_10322_MOESM1_ESM.docx]

**Seasonal Hydrochemical Characteristics of Spring Water in Southern Poland: Integrating Geochemical Modeling, Health Risk Analysis and Mitigation Strategies**

**Table 1s.** All measured parameters used for the current investigation

| **Location** | **Month** | **Sample** | **pH** | **EC (μS/cm)** | **Na**  **ppm** | **K**  **ppm** | **Ca**  **ppm** | **Mg**  **ppm** | **Cl**  **ppm** | **SO_4_**  **ppm** | **HCO_3_**  **ppm** | **NO_3_**  **ppm** | **Al**  **ppm** |
| --- | --- | --- | --- | --- | --- | --- | --- | --- | --- | --- | --- | --- | --- |
| Leśniów 1 | November | 1 | 7.36 | 384 | 5.5 | 0.5 | 90 | 1.5 | 13 | 36 | 202 | 26 | 0.005 |
| Leśniów 2 | February, | 2 | 7.03 | 399 | 5.8 | 1.5 | 89 | 3.4 | 13 | 40 | 208 | 24 | 0.005 |
| Leśniów 3 | May | 3 | 7.05 | 332 | 2.5 | 0.5 | 41 | 0.71 | 13 | 34 | 179 | 21 | 0.005 |
| Leśniów 4 | August | 4 | 6.99 | 430 | 6.9 | 2.3 | 19 | 5 | 14 | 38 | 152 | 27 | 0.062 |
| Zygmunta 1 | November | 5 | 7.13 | 359 | 9.7 | 0.5 | 81 | 0.73 | 23 | 16 | 198 | 14 | 0.005 |
| Zygmunta 2 | February, | 6 | 6.95 | 421 | 13 | 0.5 | 81 | 2.6 | 28 | 23 | 201 | 13 | 0.005 |
| Zygmunta 3 | May | 7 | 6.95 | 329 | 8.6 | 0.5 | 44 | 2.1 | 25 | 16 | 181 | 9.4 | 0.005 |
| Zygmunta 4 | August | 8 | 7.03 | 401 | 11 | 0.5 | 60 | 2.1 | 24 | 22 | 144 | 14 | 0.005 |
| Halszka 1 | November | 9 | 6.8 | 416 | 3.9 | 1.1 | 96 | 6.7 | 8 | 12 | 307 | 13 | 0.005 |
| Halszka 3 | May | 10 | 7.11 | 370 | 2.1 | 0.5 | 45 | 3.6 | 8 | 12 | 283 | 9.8 | 0.005 |
| Halszka 4 | August | 11 | 7.04 | 467 | 4.6 | 1.3 | 72 | 7 | 9 | 16 | 252 | 13 | 0.005 |
| Dobro Woda 1 | November | 12 | 5.54 | 180 | 4.4 | 2.4 | 20 | 5.3 | 8 | 63 | 6 | 1.5 | 0.093 |
| Dobro Woda 2 | February, | 13 | 6.05 | 172 | 5 | 2.3 | 27 | 7.2 | 8 | 70 | 6 | 2.8 | 0.13 |
| Dobro Woda 3 | May | 14 | 5.54 | 139 | 2.9 | 0.5 | 8.9 | 3 | 8 | 66 | 12 | 3.8 | 0.025 |
| Dobro Woda 4 | August | 15 | 6.49 | 172 | 8.4 | 7 | 19 | 4.8 | 10 | 57 | 29.6 | 0.45 | 0.025 |
| Święto Woda 1 | November | 16 | 5.86 | 175 | 5.3 | 1.9 | 25 | 4.5 | 13 | 50 | 29.1 | 3 | 0.23 |
| Święto Woda 2 | February, | 17 | 5.6 | 150 | 5.1 | 1.8 | 26 | 5.4 | 9 | 44 | 20.6 | 6.7 | 0.49 |
| Święto Woda 3 | May | 18 | 5.55 | 149 | 3.5 | 0.5 | 9.2 | 2.5 | 17 | 50 | 24 | 0.45 | 0.041 |
| Święto Woda 4 | August | 19 | 6.38 | 198 | 6.3 | 1 | 70 | 3 | 20 | 54 | 22.1 | 0.94 | 0.005 |
| ZimnySztok | November | 20 | 5.54 | 208 | 7.1 | 6.5 | 24 | 4.1 | 18 | 57 | 19.5 | 13 | 0.005 |
| ZimnySztok | February, | 21 | 6.05 | 217 | 9 | 5.9 | 32 | 6.8 | 18 | 61 | 33.3 | 11 | 0.005 |
| ZimnySztok | May | 22 | 5.11 | 184 | 3.1 | 2.3 | 8 | 1.5 | 16 | 59 | 18 | 7.9 | 0.005 |
| ZimnySztok | August | 23 | 5.34 | 214 | 4.7 | 2.4 | 15 | 5 | 17 | 56 | 6 | 12 | 0.027 |
| **Parametr** |  | 1 | **Pb**  **ppm** | **Cd**  **ppm** | **Cr**  **ppm** | **Hg**  **ppm** | **Zn**  **ppm** | **Fe**  **ppm** | **Mn**  **ppm** | **Ni**  **ppm** | **Cu ppm** | **Sr**  **ppm** |  |
| Leśniów 1 | November | 2 | 0.002 | 0.00025 | 0.0015 | 0.031 | 0.0025 | 0.002 | 0.0005 | 0.002 | 0.002 | 0.066 |  |
| Leśniów 2 | February, | 3 | 0.002 | 0.00025 | 0.0015 | 0.00012 | 0.022 | 0.002 | 0.006 | 0.002 | 0.002 | 0.066 |  |
| Leśniów 3 | May | 4 | 0.002 | 0.00025 | 0.0015 | 0.00012 | 0.0025 | 0.002 | 0.0005 | 0.002 | 0.002 | 0.028 |  |
| Leśniów 4 | August | 5 | 0.002 | 0.00025 | 0.0015 | 0.00011 | 0.009 | 0.11 | 0.027 | 0.002 | 0.002 | 0.22 |  |
| Zygmunta 1 | November | 6 | 0.002 | 0.00025 | 0.0015 | 0.00005 | 0.0025 | 0.002 | 0.0005 | 0.002 | 0.002 | 0.044 |  |
| Zygmunta 2 | February, | 7 | 0.002 | 0.00025 | 0.0015 | 0.00013 | 0.016 | 0.041 | 0.005 | 0.002 | 0.002 | 0.071 |  |
| Zygmunta 3 | May | 8 | 0.002 | 0.00025 | 0.0015 | 0.00005 | 0.015 | 0.002 | 0.004 | 0.002 | 0.002 | 0.062 |  |
| Zygmunta 4 | August | 9 | 0.002 | 0.00025 | 0.0015 | 0.00014 | 0.0025 | 0.013 | 0.003 | 0.002 | 0.002 | 0.11 |  |
| Halszka 1 | November | 10 | 0.002 | 0.00025 | 0.0015 | 0.00005 | 0.0025 | 0.002 | 0.0005 | 0.002 | 0.002 | 0.15 |  |
| Halszka 3 | May | 11 | 0.002 | 0.00025 | 0.0015 | 0.00018 | 0.0025 | 0.002 | 0.0005 | 0.002 | 0.023 | 0.072 |  |
| Halszka 4 | August | 12 | 0.002 | 0.00025 | 0.0015 | 0.00005 | 0.0025 | 0.01 | 0.004 | 0.002 | 0.002 | 0.26 |  |
| Dobro Woda 1 | November | 13 | 0.002 | 0.00025 | 0.0015 | 0.00005 | 0.023 | 0.042 | 0.019 | 0.011 | 0.002 | 0.18 |  |
| Dobro Woda 2 | February, | 14 | 0.002 | 0.00025 | 0.0015 | 0.00005 | 0.047 | 0.11 | 0.033 | 0.006 | 0.011 | 0.19 |  |
| Dobro Woda 3 | May | 15 | 0.002 | 0.00025 | 0.0015 | 0.00005 | 0.012 | 0.002 | 0.006 | 0.002 | 0.002 | 0.079 |  |
| Dobro Woda 4 | August | 16 | 0.002 | 0.00025 | 0.0015 | 0.00005 | 0.0025 | 0.017 | 0.007 | 0.002 | 0.002 | 0.16 |  |
| Święto Woda 1 | November | 17 | 0.002 | 0.00025 | 0.0015 | 0.00005 | 0.048 | 0.2 | 0.066 | 0.005 | 0.002 | 0.15 |  |
| Święto Woda 2 | February, | 18 | 0.002 | 0.00025 | 0.0015 | 0.00005 | 0.1 | 0.33 | 0.095 | 0.007 | 0.007 | 0.13 |  |
| Święto Woda 3 | May | 19 | 0.002 | 0.00025 | 0.0015 | 0.00005 | 0.007 | 0.036 | 0.004 | 0.002 | 0.002 | 0.061 |  |
| Święto Woda 4 | August | 20 | 0.002 | 0.00025 | 0.0015 | 0.00005 | 0.0025 | 0.012 | 0.003 | 0.002 | 0.002 | 0.14 |  |
| ZimnySztok 1 | November | 21 | 0.002 | 0.00025 | 0.0015 | 0.00005 | 0.0025 | 0.002 | 0.007 | 0.002 | 0.002 | 0.11 |  |
| ZimnySztok 2 | February, | 22 | 0.002 | 0.002 | 0.00025 | 0.0015 | 0.019 | 0.073 | 0.014 | 0.002 | 0.01 | 0.13 |  |
| ZimnySztok 3 | May | 23 | 0.002 | 0.002 | 0.00025 | 0.0015 | 0.019 | 0.002 | 0.003 | 0.002 | 0.002 | 0.036 |  |
| ZimnySztok 4 | August | 1 | 0.002 | 0.002 | 0.00025 | 0.0015 | 0.008 | 0.019 | 0.008 | 0.002 | 0.002 | 0.22 |  |
